# Skeletal muscle–targeted delivery of Fgf6 protects mice from diet-induced obesity and insulin resistance

**DOI:** 10.1172/jci.insight.149969

**Published:** 2021-10-08

**Authors:** Bo Xu, Caizhi Liu, Hong Zhang, Rong Zhang, Mengyang Tang, Yan Huang, Li Jin, Lingyan Xu, Cheng Hu, Weiping Jia

**Affiliations:** 1 Shanghai Diabetes Institute, Shanghai Key Laboratory of Diabetes Mellitus, Shanghai Clinical Center for Diabetes, Shanghai Jiao Tong University Affiliated Sixth People’s Hospital, Shanghai, China.; 2 Department of Endocrinology and Metabolism, Fengxian Central Hospital Affiliated to the Southern Medical University, Shanghai, China.; 3 Shanghai Key Laboratory of Regulatory Biology, Institute of Biomedical Sciences and School of Life Sciences, East China Normal University, Shanghai, China.

**Keywords:** Metabolism, Muscle Biology, Obesity, Skeletal muscle

## Abstract

Obesity, a major health care issue, is characterized by metabolic abnormalities in multiple tissues, including the skeletal muscle. Although dysregulation of skeletal muscle metabolism can strongly influence the homeostasis of systemic energy, the underlying mechanism remains unclear. We found promoter hypermethylation and decreased gene expression of fibroblast growth factor 6 (*FGF6*) in the skeletal muscle of individuals with obesity using high-throughput sequencing. Reduced binding of the cyclic AMP responsive element binding protein-1 (CREB1) to the hypermethylated cyclic AMP response element, which is a regulatory element upstream of the transcription initiation site, partially contributed to the downregulation of *FGF6* in patients with obesity. Overexpression of *Fgf6* in mouse skeletal muscle stimulated protein synthesis, activating the mammalian target of rapamycin pathway, and prevented the increase in weight and the development of insulin resistance in high-fat diet–fed mice. Thus, our findings highlight the role played by *Fgf6* in regulating skeletal muscle hypertrophy and whole-body metabolism, indicating its potential in strategies aimed at preventing and treating metabolic diseases.

## Introduction

The proportion of adults with a body mass index of 25 kg/m^2^ or greater has substantially increased worldwide in the years between 1980 and 2013 (1). The high prevalence of obesity and its close correlation to diseases, such as insulin resistance, type 2 diabetes mellitus (T2DM), and cardiovascular diseases, make obesity a major public health concern (2, 3). Skeletal muscle, comprising approximately 40% of total body mass in mammals, plays a critical role in the regulation of total body mass and the homeostasis of systemic metabolism (4); it also accounts for a majority of insulin-stimulated glucose disposal in humans (5, 6). A growing body of evidence indicates that insulin resistance in skeletal muscles is strongly associated with several metabolic syndromes and T2DM (7, 8). Saturated fatty acids and hyperglycemia have been reported to induce a decline in the mass as well as function of skeletal muscle, further aggravating metabolic abnormalities (9, 10). In contrast, physical exercise not only consumes glucose and fatty acids but also facilitates mitochondrial biogenesis, fiber remodeling or transformation, antioxidant defense mechanisms, and muscle hypertrophy, enhancing metabolic fitness (11). However, the underlying mechanism between skeletal muscle and metabolic disturbance has not been fully elucidated.

Although it is well established that there exists a genetic predisposition toward developing obesity and diabetes, an increasing number of studies have suggested the important role of epigenetic modifications in the complex interplay between genes and environment (12, 13). DNA methylation is one of the most extensively studied epigenetic modifications and almost exclusively occurs on the cytosine residues of CpG dinucleotides. Epigenome-wide association studies and other investigations have reported alterations in DNA methylation in cases of obesity, T2DM, and several metabolic syndromes, linking DNA methylation to the development or progression of metabolic disorders (13–16). Romain Barrès et al. (2009) reported hypermethylation of the PPARγ coactivator-1α (*PPARGC1A*) promoter in the skeletal muscles of patients with T2DM, concomitant with reduced mitochondrial content (17). Three years later, they reported a decrease in whole-genome methylation in skeletal muscle biopsies obtained from healthy men and women after acute exercise. Methylation of the promoter regions of genes controlling whole-body energy and glucose homeostasis, including *PPARGC1A*, *PDK4*, and *PPARD*, decreased, which was accompanied by upregulation of transcripts (18). The findings from these studies suggest a strong connection between DNA methylation in skeletal muscle and energy metabolism.

We performed high-throughput screening in skeletal muscle specimens from normal-weight individuals and participants with obesity in this study to better understand the molecular mechanism of metabolic abnormalities in skeletal muscle of patients with obesity and provide evidence for future therapeutic strategies. Fibroblast growth factors (FGFs), including FGF1–10, FGF16–18, FGF20, and FGF22, are considered paracrine factors and are known for regulating tissue patterning and organogenesis during embryogenesis (19). However, FGF19, FGF21, and FGF23 together constitute a unique endocrine-like subfamily and function by regulating critical metabolic processes in a variety of tissues (20, 21). Unlike other FGFs detected in developing skeletal muscle, *Fgf6* exhibits a restricted expression profile predominantly in the myogenic lineage (22, 23), indicating its role in myogenesis. *Fgf6* expression was also recently reported to be dramatically induced by exercise training in adipose tissue; it was identified as a potent inducer of uncoupling protein-1 expression in adipocytes and preadipocytes, modulating systemic energy metabolism (24). In this study, we identified downregulation of *FGF6* transcript levels and hypermethylation of its promoter region in individuals with obesity after we analyzed sequencing data of skeletal muscle specimens. Given the lack of understanding about the functions of *FGF6* in skeletal muscle metabolism, we aimed to investigate epigenetic modifications in the *FGF6* promoter region and its effects in skeletal muscle specimens of individuals with obesity. Furthermore, we examined the effects of *Fgf6* overexpression in mice fed a high-fat diet.

## Results

### The FGF6 promoter is hypermethylated and mRNA levels are decreased in the skeletal muscle of individuals with obesity.

**Figure 1:**
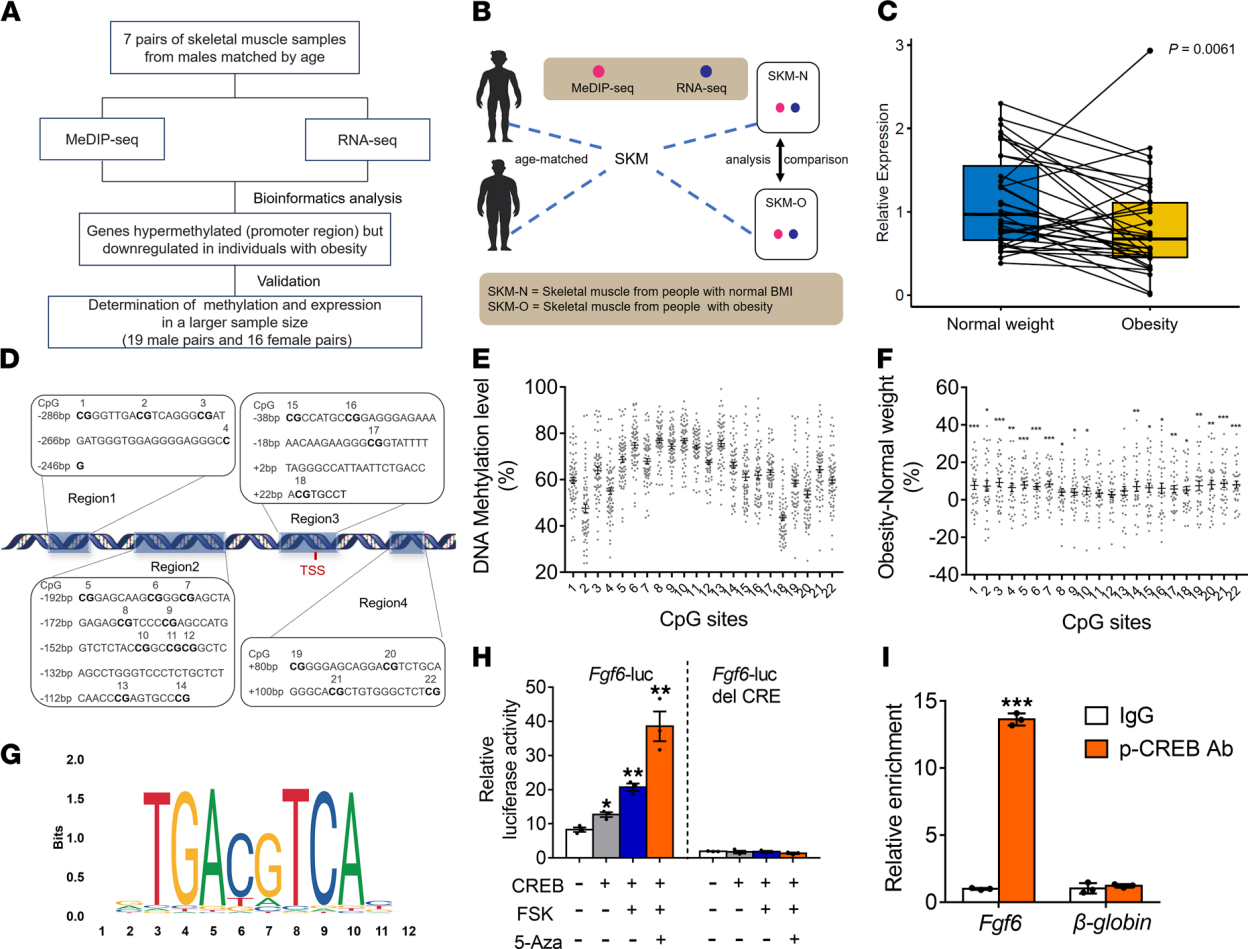
*FGF6* promoter is hypermethylated in the skeletal muscle of participants with obesity, leading to the inhibition of CREB1-mediated transcriptional activation. (**A**) Flow chart of the experimental design to screen differentially expressed genes in the skeletal muscle of normal-weight individuals and participants with obesity. (**B**) Abridged general view of the 7 pairs of age-matched skeletal muscle specimens from men subjected to MeDIP-Seq, RNA-Seq, and further analysis. (**C**) Comparison of relative mRNA levels of *FGF6* in 35 pairs of age-matched skeletal muscle specimens from normal-weight individuals and participants with obesity. (**D**) Sequences of the *FGF6* promoter region selected for validation by pyrosequencing in 35 pairs of skeletal muscle tissues. (**E**) Mean methylation level of each CpG site (*n* = 35 pairs). (**F**) Variation of methylation levels between skeletal muscles from individuals with obesity and their matched normal-weight controls (*n* = 35 pairs). (**G**) CRE in the promoter region of *FGF6*. (**H**) Luciferase activity of the WT *Fgf6* reporter or the mutant *Fgf6* reporter, containing the CRE deletion (*Fgf6*-luc del CRE), in HEK293T cells transiently expressing either an empty vector or CREB1-expressing vector, treated with forskolin (FSK) or 5-Aza; *n* = 3 per group. (**I**) ChIP analysis of phosphorylated CREB1 (p-CREB1) binding on the putative CRE identified in the *Fgf6* promoter or on the β*-globin* promoter in mouse gastrocnemius muscle (*n* = 3 per group). Data information: The box plot represents data from the first quartile to the third quartile. The second quartile is the median of the data (**C**). Other results (**E**, **F**, **H**, and **I**) are represented as mean ± standard error of mean. Statistical analysis was done using paired and unpaired Student’s *t* tests or Wilcoxon’s signed rank sum tests where appropriate. *P < 0.05, **P < 0.01, ***P < 0.001.

To investigate the effects of obesity on skeletal muscle in human beings, we performed a genome-wide DNA methylation sequencing analysis in conjunction with transcriptomic analysis and further verified the results in a larger sample cohort (Figure 1A). First, 7 pairs of age-matched skeletal muscle samples from men were subjected to methylated DNA immunoprecipitation sequencing (MeDIP-Seq) and RNA sequencing (RNA-Seq; Figure 1B and Supplemental Table 1; supplemental material available online with this article; https://doi.org/10.1172/jci.insight.149969DS1), revealing that both DNA methylation and transcriptomic profiles were altered in the skeletal muscles of people with obesity (Supplemental Figures 1 and 2). Among the differential genes, 13 genes exhibited a hypermethylated promoter region and decreased transcript expression in the skeletal muscle of participants with obesity (Supplemental Table 2). Of these, we selected 3 genes for further analysis that are mainly expressed in the skeletal muscle (*MYH3*, *FGF6*, and *XIRP1*), and *YTHDC1*, which encodes an N^6^-methyladenosine reader protein, because of its important role in m^6^A methylation-mediated regulation of adipogenesis and liver steatosis (25, 26). The expressions of these genes were validated in 35 pairs (19 male pairs and 16 female pairs) of age-matched skeletal muscle specimens (Supplemental Table 3). Quantitative reverse transcription PCR (qPCR) results showed that skeletal muscle samples from individuals with obesity exhibited lower *FGF6* levels than those from normal-weight participants (Figure 1C). The expression levels of the other 3 genes were not affected by obesity (Supplemental Figure 3, A–C). Next, pyrosequencing was utilized to evaluate the DNA methylation levels in the promoter region of *FGF6*. Sequences from –286 bp upstream to +119 bp downstream from the transcriptional start site (TSS) were amplified, and the DNA methylation levels were estimated. Details of the nucleotide sequences and the specific location of each CpG site are presented in Figure 1D. Among the 22 CpG sites analyzed, the average methylation levels ranged from 43.58% to 76.95%, with CpG 2 and CpG 18 exhibiting a stark reduction in methylation compared with the methylation levels of surrounding sites (47.64% for CpG 2 and 43.58% for CpG 18; Figure 1E and Supplemental Table 4). Further, people with obesity showed significantly higher levels of DNA methylation than normal-weight individuals in a majority of the 22 CpG sites (Figure 1F and Supplemental Table 5).

### Increased methylation of the cAMP responsive element inhibits the transcription of FGF6.

To further elucidate the transcriptional regulation of *FGF6*, the JASPAR database was used to identify possible trans-acting factors and their corresponding binding sequences in the promoter region of *FGF6*. The sequences analyzed for DNA methylation, as shown in Figure 1D, were scanned for promoter binding sites, and the top 10 results contained 2 cis-acting elements, the cAMP responsive element (CRE; Figure 1G) and the enhancer box (E-box; Supplemental Figure 3D), which were composed of CpG 2 and CpG 18, respectively (Supplemental Table 6). CREB1 was predicted to bind to the CRE (–283 bp to –272 bp from the TSS), while basic helix-loop-helix proteins, including MXI1, MYC, MNT, and MAX, showed high affinity toward the E-box (+17 bp to +29 bp downstream of the TSS). The relatively low methylation levels of CpG 2 and CpG 18 (Figure 1E and Supplemental Table 4) further strengthened the reliability of the predictions. Next, luciferase reporter assays using the *Fgf6* promoter region, consisting of the CRE and E-box, were performed. MYC was selected as a candidate to be overexpressed together with CREB1, as MYC activities are modulated by a network of nuclear basic helix-loop-helix leucine zipper proteins, with MYC-MAX complexes activating transcription and MXI1 and MNT acting as antagonists of MYC. The results showed that overexpression of CREB1 moderately increased the transcription activity of HEK293T cells transfected with the CRE luciferase reporter (Figure 1H). The combination of CREB1 overexpression and forskolin, an inducer of intracellular cAMP generation, significantly increased the reporter activity of luciferase, which was further increased with the addition of 5-Aza-2′-Deoxycytidine (5-Aza), a DNA-hypomethylating agent (Figure 1H). This result suggests that binding of phosphorylated CREB1 to the CRE promotes the transcription of *Fgf6*, and the methylation of CpG in the CRE limits this binding. In addition, the luciferase signal in the HEK293T cells transfected with a mutant *Fgf6* reporter without the MYC binding site after MYC overexpression was slightly weaker than in those transfected with the WT *Fgf6* reporter (Supplemental Figure 3E). Next, using ChIP-qPCR, we confirmed that there was substantial enrichment of CREB1 binding, but not of MYC, at the *Fgf6* promoter region in the skeletal muscle of mice (Figure 1I and Supplemental Figure 3F). Therefore, we can conclude that the decrease in *FGF6* mRNA in obese skeletal muscle is affected, at least in part, by the elevated methylation level of CpG in the CRE.

### Overexpression of Fgf6 in skeletal muscle promotes hypertrophy.

**Figure 2:**
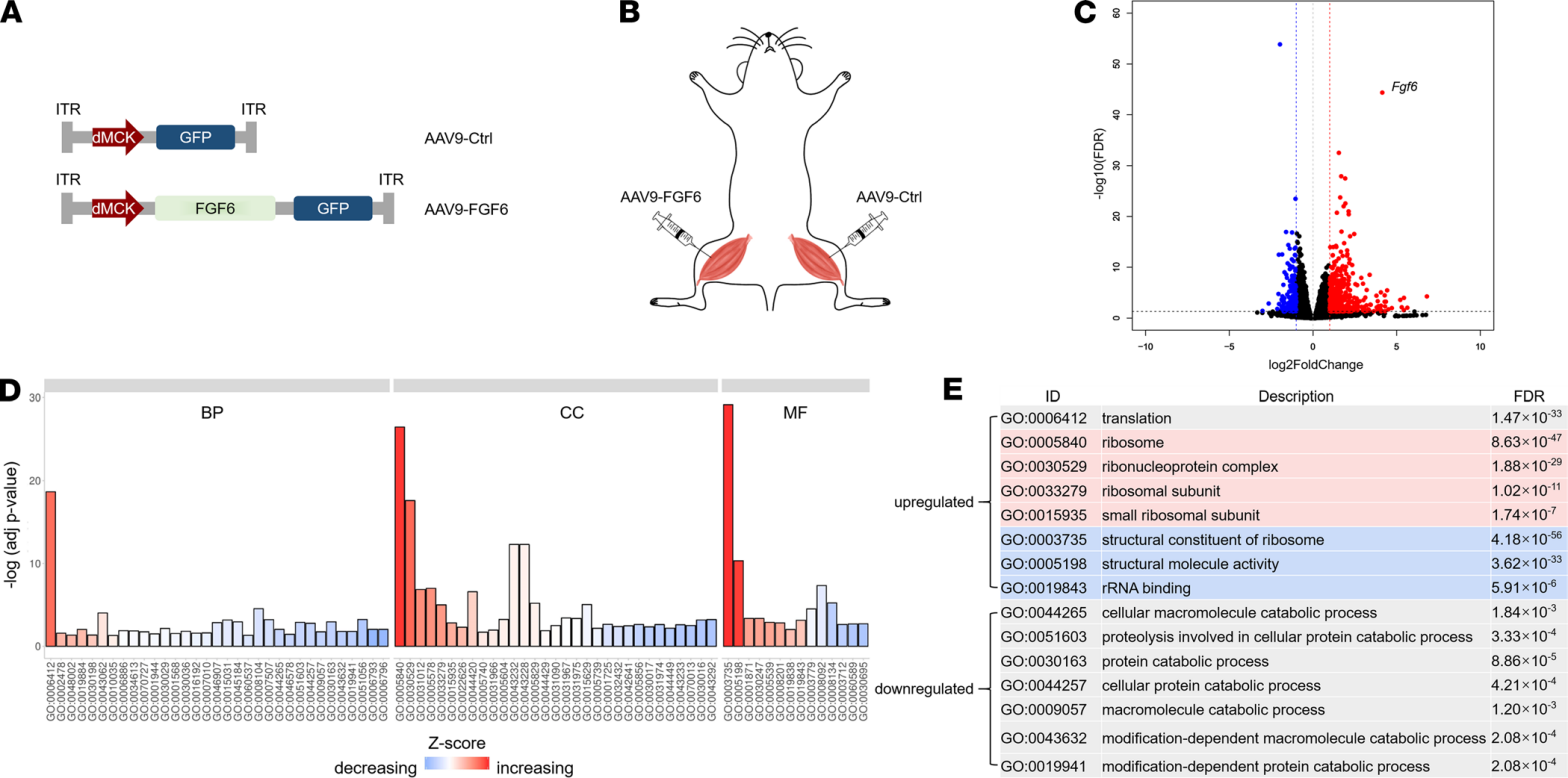
AAV9-mediated *Fgf6* overexpression for 1 month in the skeletal muscle of mice results in a net increase in protein synthesis. (**A**) Diagrammatic representation of AAV9-FGF6 and the empty viral vector AAV9-control (Ctrl) used in the study. ITR, inverted terminal repeat. (**B**) Illustration of the experimental setup: AAV9-FGF6 was injected into the gastrocnemius (gastroc) muscle of 1 leg, while the contralateral muscle was injected with AAV9-Ctrl. (**C**) Volcano plot showing the differentially expressed genes (DEGs) in the gastroc muscle 1 month after AAV9-FGF6 injection (*n* = 3). Genes with adjusted *P* value (*P*_adj_) less than 0.05 were considered DEGs (multiple comparisons were corrected using the Benjamini–Hochberg procedure). Red and blue dots represent genes that were upregulated (log_2_ fold change ≥ 1) or downregulated (log_2_ fold change ≤ –1), respectively. (**D**) GO analysis of DEGs with log_2_ fold change ≥ 1 or ≤ –1. BP, biological process; CC, cellular component; MF, molecular function. (**E**) Representative GO terms related to protein synthesis and catabolism. Gray, red, and blue background colors represent BP, CC, and MF, respectively.

*Fgf6* in the adipose tissue has recently emerged as a potential therapeutic target in metabolic diseases (24), and we found that its expression was suppressed in the skeletal muscles of individuals with obesity. Given that the skeletal muscle is a key metabolic organ, we explored the role of *Fgf6* overexpression there. To achieve this, we utilized a skeletal muscle–specific promoter (d*MCK*), which has been reported to be suitable for adeno-associated virus (AAV) vectors in muscle-directed gene therapy studies (Figure 2A) (27). Mice were injected with AAV serotype 9–FGF6 (AAV9-FGF6) in the gastrocnemius (gastroc) muscle of 1 leg, while the contralateral muscle was injected with an empty viral vector (Figure 2B). RNA-Seq was performed 1 month after injection, and a total of 2772 differentially expressed genes were identified in the *Fgf6* overexpression samples (1288 upregulated and 1484 downregulated; Figure 2C). Gene ontology (GO) analysis revealed that these differentially expressed genes were significantly enriched for translation, ribosome, and catabolic process, suggesting increased protein synthesis as well as decreased proteolysis in the skeletal muscle (Figure 2, D and E). Subsequently, Kyoto Encyclopedia of Genes and Genomes analysis also revealed ribosome (mmu03010) as the top enriched pathway (FDR = 2.59 × 10^–40^).

**Figure 3:**
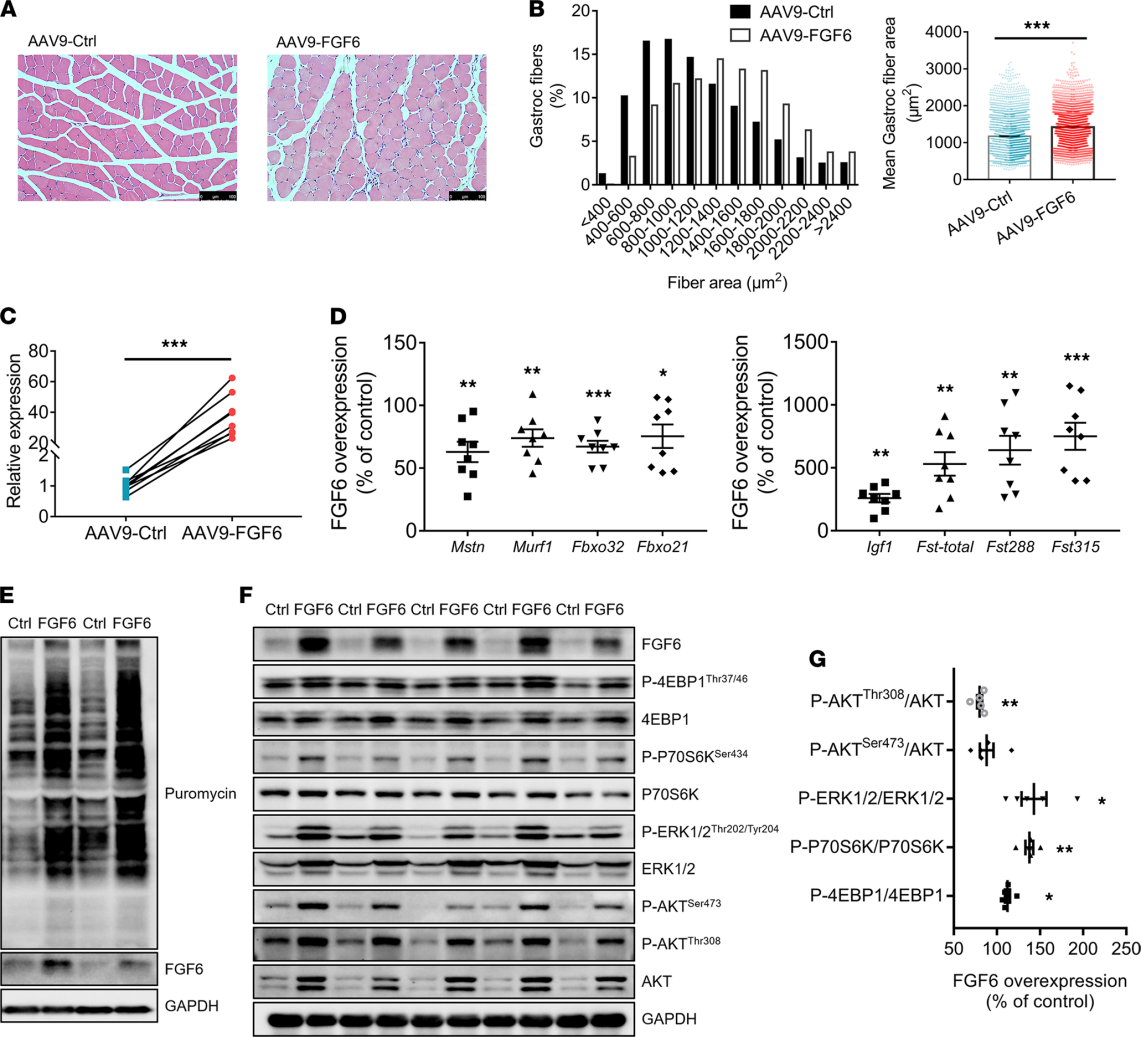
*Fgf6* promotes skeletal muscle hypertrophy. (**A**) Representative images of H&E-stained muscles. Scale bars: 100 μm. (**B**) Frequency distribution of cross-sectional area (CSA) and mean fiber area of the stained muscle fibers (*n* = 4). (**C**) Relative mRNA level of *Fgf6* determined by qPCR in gastroc muscles injected with AAV9-Ctrl or AAV9-FGF6 (*n* = 8). (**D**) Alterations of relative mRNA levels of genes relevant to muscle atrophy (*Mstn*, *Murf1*, *Fbxo32*, and *Fbxo21*) and hypertrophy (*Igf1*, *Fst-total*, *Fst288*, and *Fst315*) after *Fgf6* overexpression in gastroc muscles (*n* = 8). *Mstn*, myostatin; *Murf1*, muscle ring finger 1; *Igf1*, insulin like growth factor 1; *Fst*, follistatin. (**E**) Western blotting analysis depicting the changes in protein synthesis rates (tracked using SUnSET assay) and FGF6 in gastroc muscles following AAV9-FGF6 injection (*n* = 2). SUnSET, surface sensing of translation. (**F**) Representative images of Western blotting analysis of the mammalian target of rapamycin signaling proteins in lysates from skeletal muscles injected with AAV9-Ctrl or AAV9-FGF6 (*n* = 5). (**G**) Comparison of the ratio of phosphorylated protein to the total protein in the lysates of skeletal muscles with or without *Fgf6* overexpression. Integrated density of phosphorylated protein (**F**) band was divided by that of the total protein (**F**) band (*n* = 5). Data information: Results are represented as mean ± standard error of mean. Unpaired and paired Student’s *t* test comparisons were conducted. *P < 0.05, **P < 0.01, ***P < 0.001.

Although the mass of the gastroc muscle injected with AAV9-FGF6 remained unchanged (Supplemental Figure 4A), we found that *Fgf6*-treated gastroc muscles showed a shift toward larger myofibers as compared with that of the control muscle (Figure 3, A and B), based on CSA measurements of H&E-stained sections, indicating the occurrence of skeletal muscle hypertrophy. qPCR analysis confirmed the overexpression of *Fgf6* (approximately 36.9-fold; Figure 3C) and showed that the expression of genes critical for muscle atrophy and hypertrophy (28–30) was influenced by *Fgf6* expression levels. As shown in Figure 3D, atrophy-related genes, including *Mstn*, *Murf1*, *Fbxo32*, and *Fbxo21*, showed decreased expression in muscles overexpressing *Fgf6* compared with that in control muscles. Meanwhile, the mRNA levels of genes involved in muscle proteostasis and growth (31–33), namely, *Igf1*; *Fst*-*total*; and its 2 main isoforms, *Fst288* and *Fst315*, were increased in the *Fgf6*-overexpressing muscles compared with those in control muscles (Figure 3D). In addition, the abundance of 45S pre-rRNA, which is necessary for hypertrophy (34), showed an approximately 1.3-fold increase in muscles overexpressing *Fgf6* compared with that in control muscles (Supplemental Figure 4B). Analysis of puromycin-labeled peptides further confirmed the enhanced protein synthesis in *Fgf6*-overexpressing muscles (Figure 3E). Together, these results indicate that protein synthesis of skeletal muscle is elevated by *Fgf6*.

It is well established that protein synthesis is tightly regulated by the downstream effectors of the mammalian target of rapamycin (mTOR) signaling pathway (35). We found that overexpression of *Fgf6* significantly stimulated the phosphorylation of both 4EBP1 and P70S6K, although 4EBP1 and P70S6K were also upregulated simultaneously (Figure 3, F and G). It has been previously reported that AKT and ERK1/2 can activate mTOR (36, 37), and not surprisingly, we observed increased p-ERK1/2^Thr202/Tyr204^, p-AKT^Ser473^, and p-AKT^Thr308^ levels in AAV9-FGF6–injected muscles (Figure 3F). The ratio of p-ERK1/2^Thr202/Tyr204^ to ERK1/2 was also elevated in the AAV9-FGF6–injected muscles, in spite of the increased expression of total ERK1/2 (Figure 3G). Although the p-AKT^Ser473^/AKT ratio remained unchanged, the p-AKT^Thr308^/AKT ratio decreased because of the stark upregulation of total AKT (Figure 3G) in the AAV9-FGF6–injected muscles. These results suggest that *Fgf6* may target the mTOR pathway through phosphorylated AKT and ERK1/2, ultimately activating P70S6K and 4EBP1.

### Fgf6 plays a long-term role in stimulating protein synthesis, independent of dietary intake.

**Figure 4:**
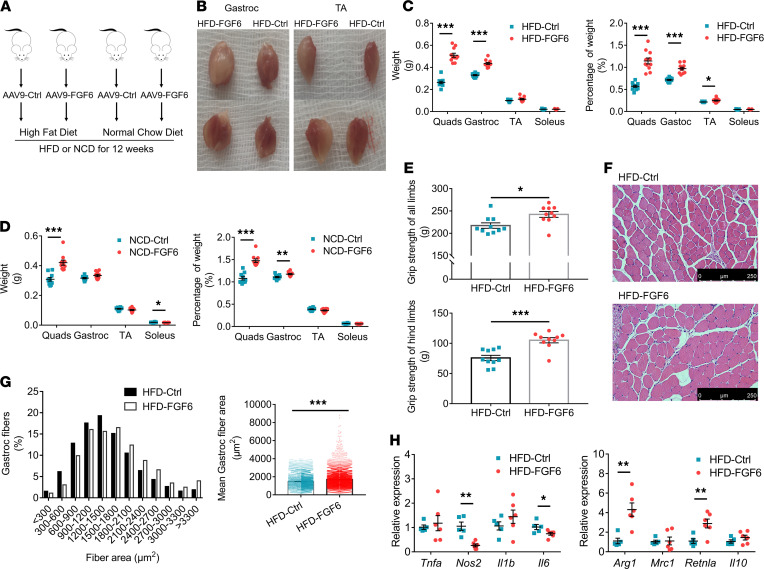
Long-term (12 weeks) *Fgf6* overexpression increases muscle weight and function in diet-induced obese mice. (**A**) Graphical illustration of mouse groups based on AAV injection and diet. (**B**) Photographic images of gastroc and tibialis anterior (TA) muscles in the HFD-fed group injected with AAV9-Ctrl or AAV9-FGF6. (**C**) Mass of quadriceps (quads), gastroc, TA, and soleus muscles in HFD-fed mice (*n* = 11 per group). Percentage of muscle weight was calculated by dividing muscle weight by the corresponding body weight and then multiplying by 100. (**D**) Measurement of muscle weight of quads, gastroc, TA, and soleus muscles and the calculated percentage of muscle weight in NCD-fed mice (*n* = 11 per group). (**E**) Function analysis of grip strength of HFD-fed mice (*n* = 10 per group). (**F**) Representative images of H&E-stained gastroc muscles in HFD-fed mice. Scale bars, 250 μm. (**G**) Frequency distribution of CSA and mean fiber area of the stained muscle fibers in HFD-fed mice (*n* = 4 per group). (**H**) Expression levels of M1 macrophage markers (*Tnfa*, *Nos2*, *Il1b*, and *Il6*) and M2 macrophage markers (*Arg1*, *Mrc1*, *Retnla*, and *Il10*) in gastroc muscles of HFD-fed mice injected with AAV9-Ctrl (*n* = 5) or AAV9-FGF6 (*n* = 6). Data information: Results are represented as mean ± standard error of mean. Statistical analysis was done using unpaired Student’s *t* tests. *P < 0.05, **P < 0.01, ***P < 0.001.

To identify the long-term effects of *Fgf6* in the skeletal muscle, we assigned mice into 4 groups according to the AAV treatment (AAV9-FGF6 or AAV9-Ctrl) and diet (high-fat diet, HFD; or normal chow diet, NCD), namely HFD-FGF6, HFD-Ctrl, NCD-FGF6, and NCD-Ctrl. After AAV injection (quadriceps [quads], gastroc, and tibialis anterior [TA] muscles on both sides), mice were put on an HFD or NCD for 12 weeks (Figure 4A). In the HFD group, mice injected with AAV9-FGF6 exhibited visibly larger muscles compared with those in the mice injected with AAV9-Ctrl (Figure 4B). Correspondingly, weights of the quads and gastroc muscles increased significantly with *Fgf6* overexpression, and so did the ratio of quads, gastroc, or TA to the body weight (Figure 4C). Further, mice fed with an NCD showed a similar phenotype in muscle weight as that in the HFD group, with NCD-FGF6 mice presenting heavier quads than the NCD-Ctrl mice (Figure 4D). In addition, we observed that the HFD-FGF6 mice had better muscle strength, as assessed by a grip test (Figure 4E), compared with that in the HFD-Ctrl mice; the hypertrophic effect of *Fgf6* was also present in the HFD group (Figure 4, F and G). Strikingly, both H&E and Oil Red O staining showed increased fat deposition in the gastroc muscle of the HFD-FGF6 mice compared with the HFD-Ctrl mice (Supplemental Figure 4, C and D). Meanwhile, triglyceride (TG) levels in the skeletal muscle of HFD-FGF6 mice were significantly higher than in that of the HFD-Ctrl mice (Supplemental Figure 4E). However, no increase in proinflammatory cytokines was observed; instead, *Nos2* and *Il6* decreased in gastroc muscles injected with AAV-FGF6 in the HFD group. Furthermore, the markers of M2 macrophages, recognized as an antiinflammatory or reparatory subset of macrophages, including *Arg1* and *Retnla*, were increased in HFD-FGF6 mice compared with those in the HFD-Ctrl mice (Figure 4H).

**Figure 5:**
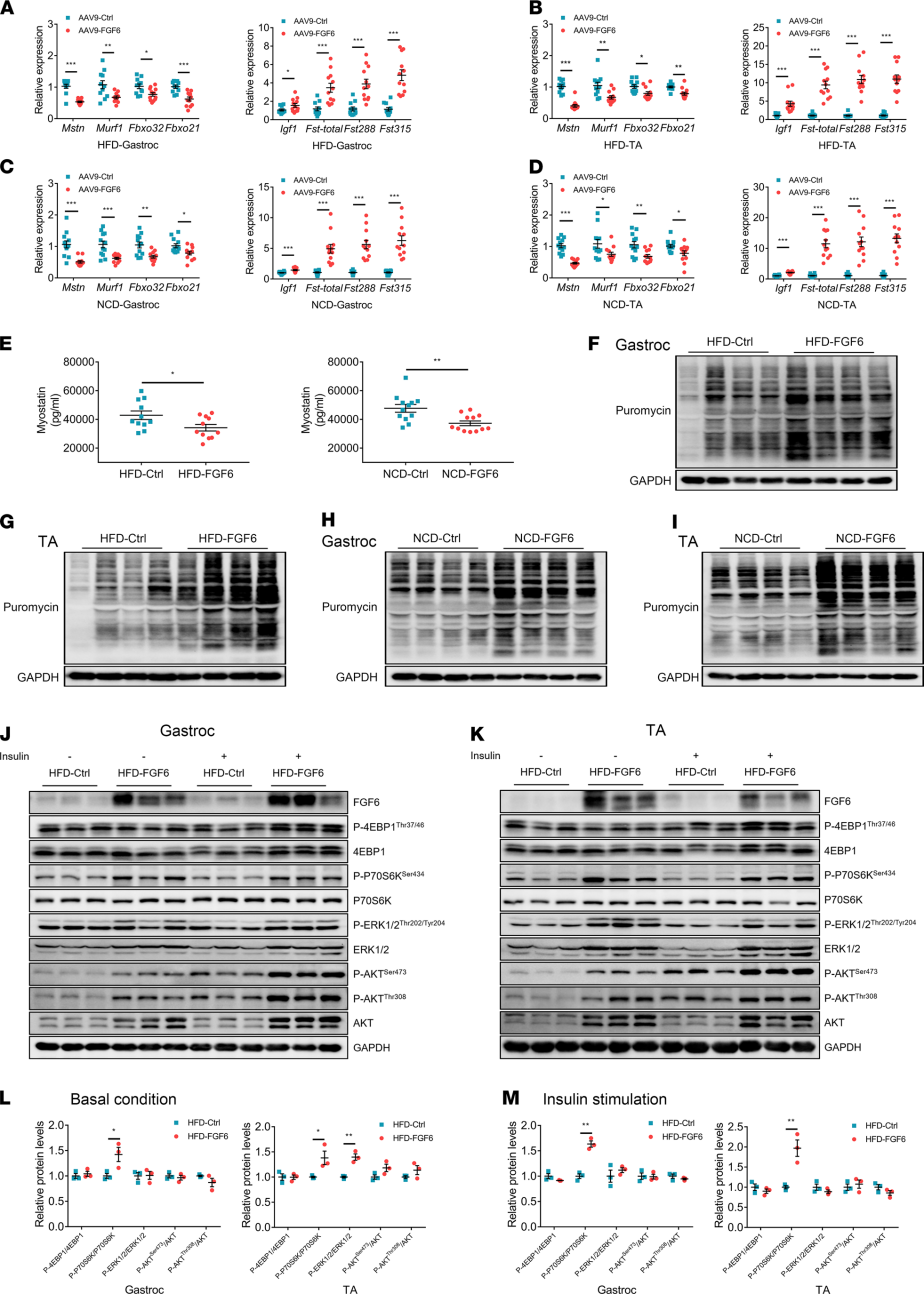
*Fgf6* persistently promotes protein synthesis for 12 weeks in skeletal muscle. (**A**–**D**) mRNA levels of atrophy-related (*Mstn*, *Murf1*, *Fbxo32*, and *Fbxo21*) and hypertrophy-related (*Igf1*, *Fst-total*, *Fst288*, and *Fst315*) genes in the gastroc muscles of HFD-fed mice (**A**), TA muscles of HFD-fed mice (**B**), gastroc muscles of NCD-fed mice (**C**), and TA muscles of NCD-fed mice (**D**) measured using qPCR (*n* = 11–12 per group). (**E**) Myostatin levels in the serum of AAV9-Ctrl– and AAV9-FGF6–injected mice fed an HFD or NCD (*n* = 11–12 per group). (**F**–**I**) Images of the Western blotting, tracked with SUnSET analysis, showing changes in protein synthesis rates in gastroc and TA muscles 12 weeks after AAV9 injection in HFD- or NCD-fed mice (*n* = 4 per group). (**J** and **K**) Western blot analysis of the mammalian target of rapamycin signaling proteins in gastroc (**J**) and TA (**K**) muscles; *n* = 3 per group. (**L** and **M**) Comparison of the ratio of phosphorylated protein to the total protein in muscles with or without *Fgf6* overexpression in basal (**L**) or insulin-stimulated (**M**) conditions. Integrated density of the phosphorylated protein (**J** or **K**) band was divided by that of the total protein (**J** or **K**) band (*n* = 3 per group). Data information: Results are represented as mean ± standard error of mean. Statistical analysis was done using unpaired Student’s *t* tests. *P < 0.05, **P < 0.01, ***P < 0.001.

Next, we determined the expression of genes associated with skeletal muscle atrophy and hypertrophy. In accordance with the results of the 1-month overexpression experiments, *Fgf6* overexpression prominently suppressed the expression of *Mstn*, *Murf1*, *Fbxo32*, and *Fbxo21* but upregulated that of *Igf1*, *Fst*-total, *Fst288*, and *Fst315* in gastroc and TA muscles compared with those in control mice in both the HFD- and NCD-fed groups (Figure 5, A–D). We further estimated myostatin levels in the serum and found a stark reduction in circulating myostatin concentrations in the AAV9-FGF6 mice compared with concentrations in AAV9-Ctrl mice, and the reduction was independent of the diet fed (Figure 5E). Additionally, the *Fgf6*-mediated increase in protein synthesis, as estimated using the nonisotopic SUnSET assay, persisted 12 weeks after AAV delivery (Figure 5, F–I).

The monthlong overexpression of *Fgf6* in the NCD mice was found to robustly augment P70S6K and 4EBP1 signals. Thus, we estimated the levels of these proteins in HFD-fed obese mice. The p-P70S6K^Ser434^, p-ERK1/2^Thr202/Tyr204^, p-AKT^Ser473^, and p-AKT^Thr308^ levels increased, regardless of insulin stimulation, in both gastroc and TA muscles of the HFD-FGF6 mice compared with the levels in HFD-Ctrl mice; however, an upregulation of p-4EBP1^Thr37/46^ in the gastroc and TA muscles of HFD-FGF6 mice was observed only after insulin stimulation (Figure 5, J and K; and Supplemental Figure 4, F and G). Moreover, *Fgf6* overexpression led to an increase in the total protein levels of 4EBP1 (observed after insulin stimulation), ERK, and AKT in both the gastroc and TA muscles of the HFD-FGF6 mice compared with the levels in HFD-Ctrl mice (Figure 5, J and K; and Supplemental Figure 4, F and G). The ratio of phosphorylated protein/total protein was then calculated, and an increase in the p-P70S6K^Ser434^/P70S6K ratio was found in both the gastroc and TA muscles of the HFD-FGF6 mice compared with the levels in HFD-Ctrl mice. In addition, *Fgf6* overexpression increased the expression and activity of ERK1/2 and AKT to a similar extent (Figure 5, L and M).

### Fgf6 overexpression in skeletal muscle reduces the mass of adipose tissue regardless of diet group and alleviates HFD-induced metabolic disturbance.

**Figure 6:**
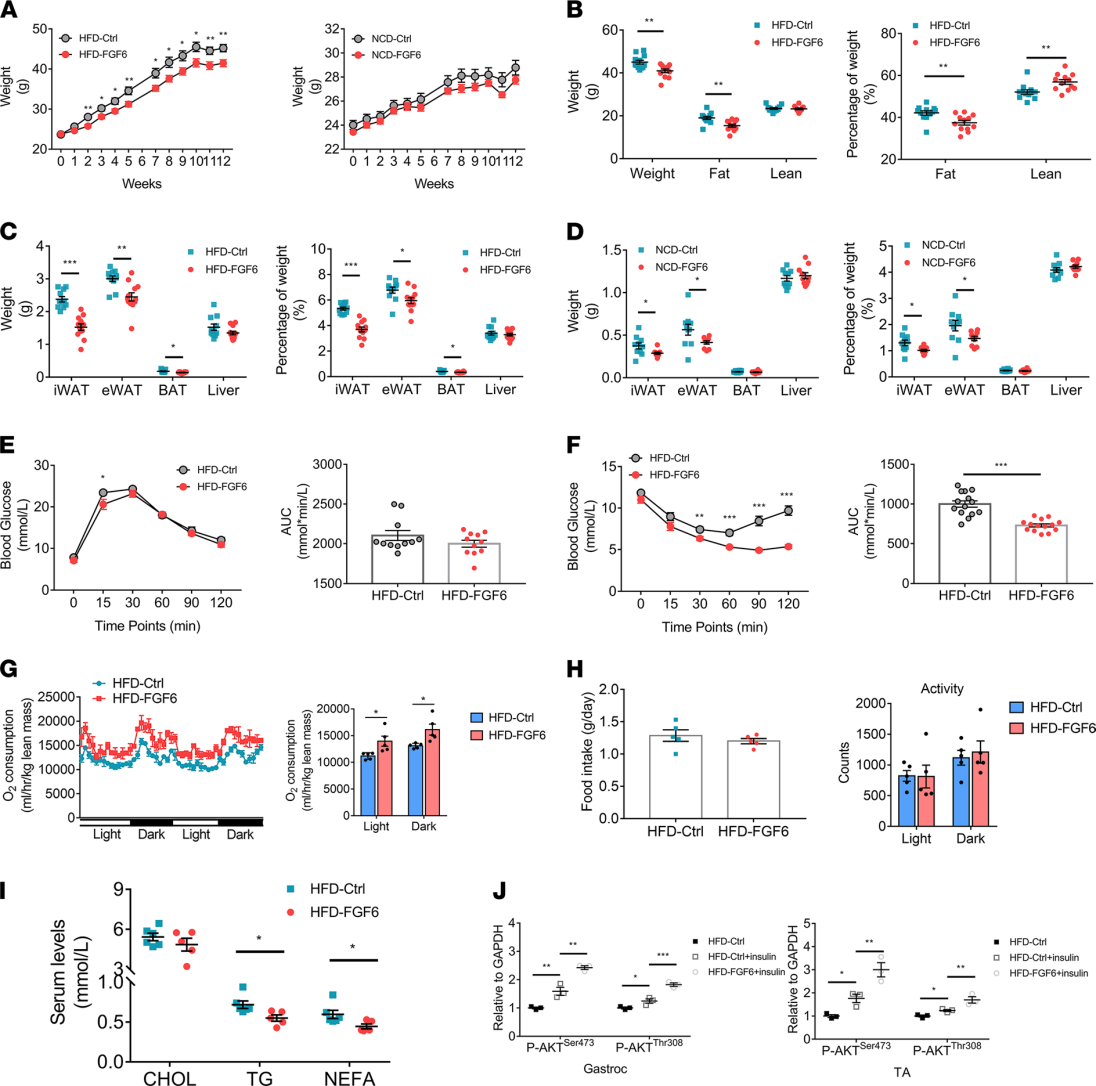
Overexpression of *Fgf6* in mice skeletal muscle prevents HFD-induced obesity and enhances whole-body metabolism. (**A**) Body weight of mice injected with AAV9-Ctrl or AAV9-FGF6, on either HFD or NCD (*n* = 12 per group). (**B**) Body composition analysis of HFD-fed mice, evaluated by EchoMRI (*n* = 12 per group). Percentage of fat or lean weight was calculated by dividing fat or lean mass by body weight, then multiplying by 100. (**C** and **D**) Weight and calculated percentage of weight of inguinal subcutaneous white adipose tissue (iWAT), epididymal white adipose tissue (eWAT), brown adipose tissue (BAT), and liver from AAV9-Ctrl–injected or AAV9-FGF6–injected HFD-fed (**C**) or NCD-fed (**D**) mice; *n* = 11–12 per group. (**E** and **F**) GTT (**E**) and ITT (**F**) in HFD-fed mice at 10 weeks and 11 weeks, respectively (*n* = 12–15 per group). AUC, area under the curve. (**G** and **H**) The whole-body energy metabolism (**G**), food intake, and activity (**H**) of HFD-Ctrl and HFD-FGF6 mice was measured in a comprehensive laboratory animal monitoring system for 48 hours (*n* = 5 per group). (**I**) Serum levels of cholesterol (CHOL), triglyceride (TG), and NEFA in HFD-fed mice with or without FGF6 overexpression (*n* = 5–6 per group). Mice were fasted overnight before blood collection. (**J**) Quantification of p-AKT^Ser473^ and p-AKT^Thr308^ in gastroc and TA muscles in response to insulin stimulation in HFD-Ctrl and HFD-FGF6 mice (*n* = 3 per group). Data information: Results are represented as mean ± standard error of mean. Statistical analysis was done using unpaired Student’s *t* tests or 1-way ANOVA correcting for multiple comparisons by controlling the FDR where appropriate. *P < 0.05, **P < 0.01, ***P < 0.001.

Enhancing the muscle mass by targeted pharmacologic or genetic regulation of muscle-regulatory molecules, such as myostatin and FST, has been reported as a promising approach to improve muscle metabolic health (32, 33, 38). Therefore, we sought to determine the role of *Fgf6* in muscle metabolism. We monitored the weight of mice mentioned in Figure 4A every week (except week 6) after the AAV injection and found that the weight gain HFD induced started to slow down 2 weeks after the injection of AAV9-FGF6. Weights of the NCD-FGF6 mice were similar to those of the NCD-Ctrl mice (Figure 6A). We determined the body composition 12 weeks after AAV9 injection and found that the HFD-FGF6 mice had lower body weight and fat mass than the HFD-Ctrl mice, though with no difference in their lean mass. However, *Fgf6* reduced body fat percentage and increased lean mass percentage in the HFD-FGF6 mice compared with those in HFD-Ctrl mice (Figure 6B). In addition to muscle mass (Figure 4, C and D), we compared the weight of adipose tissues and the liver between the *Fgf6* overexpression and control mice. Total inguinal subcutaneous white adipose tissue (iWAT), epididymal white adipose tissue (eWAT), and brown adipose tissue (BAT) as well as the ratio of iWAT, eWAT, and BAT to body weight decreased in the HFD-FGF6 mice compared with those in HFD-Ctrl mice (Figure 6C). Although there was no difference in weight between the NCD-FGF6 and NCD-Ctrl mice, the NCD-FGF6 group showed less fat, including iWAT and eWAT, compared with the control mice (Figure 6D).

To elucidate the effect of *Fgf6* on glucose and insulin tolerance, glucose tolerance test (GTT) and insulin tolerance test (ITT) were performed at weeks 10 and 11 after AAV delivery, respectively. Mice injected with AAV9-FGF6 showed a slight improvement in glucose tolerance compared with the control mice in both the HFD and NCD groups (Figure 6E and Supplemental Figure 5A). Results of the ITT indicated that overexpression of *Fgf6* in the skeletal muscle not only restored insulin sensitivity in the HFD-fed mice but also improved insulin sensitivity of the NCD-fed mice to a certain extent (Figure 6F and Supplemental Figure 5B). Metabolic cages were used to gain additional insight into the impact of *Fgf6* on whole-body metabolism and revealed an increase in O_2_ consumption, normalized to lean body mass, in the HFD-FGF6 mice compared with that in HFD-Ctrl mice (Figure 6G), with no difference in food intake and activity (Figure 6H). In addition, serum TG and nonesterified fatty acid (NEFA) levels were much lower in HFD-FGF6 mice than in HFD-Ctrl mice (Figure 6I). As both AAV9-Ctrl and AAV9-FGF6 contain sequences coding for GFP (Figure 2A), we ruled out the spillover of AAV9 infection in other tissues because there was no detection of GFP protein in the iWAT, eWAT, liver (Supplemental Figure 5C), and BAT (Supplemental Figure 5D). To decipher the target of enhanced insulin sensitivity in the HFD-FGF6 mice, we observed p-AKT in the adipose tissues (iWAT and eWAT; Supplemental Figure 5, E and F), liver (Supplemental Figure 5G), and skeletal muscles (gastroc and TA; Figure 5, J and K, and Figure 6J), supporting the upregulation of AKT phosphorylation in the skeletal muscle of *Fgf6*-treated mice. Although insulin stimulation robustly upregulated p-AKT^Ser473^ and p-AKT^Thr308^ in the gastroc and TA muscles, the levels of phosphorylated proteins further increased upon *Fgf6* overexpression. Subsequently, the effect of FGF6 on muscle mitochondrial characterization in vitro and in vivo was studied. When recombinant FGF6 was added to myotubes for 24 hours, the basal O_2_ consumption of C2C12 was increased, which depends on the role of FGF6 in promoting protein synthesis; however, the maximal respiratory capacity was unaffected (Supplemental Figure 6A). In addition, no increase in the mitochondrial density of the gastroc muscles was observed in HFD-FGF6 mice compared to that in HFD-Ctrl mice (Supplemental Figure 6B). We investigated whether AMPK can be regulated by *Fgf6* overexpression, because AMPK plays a crucial role in controlling energy homeostasis, and found that the ratio of p-AMPKα^Thr172^/AMPK was elevated in both the gastroc and TA muscles in the HFD-fed mice on treatment with AAV9-FGF6 (Supplemental Figure 6C). Simultaneously, the AMP/ATP and ADP/ATP ratios increased after AAV9-FGF6 treatment in the gastroc muscles of mice fed with an HFD (Supplemental Figure 6D).

In summary, our results suggest that *Fgf6* overexpression ameliorates insulin resistance, improves basal oxygen consumption of mitochondria, and increases the AMPK activity of skeletal muscles, enhancing systemic glucose and lipid metabolism.

## Discussion

In this study, we uncovered a pivotal role of *Fgf6* in skeletal muscle metabolism, particularly in humans with obesity and HFD-fed mice. *FGF6* was hypermethylated in the promoter region and exhibited a decline in gene expression in the skeletal muscle of individuals with obesity. We found that the hypermethylation of CRE, which inhibited the attachment of CREB1, contributed to the *FGF6* expression decline. Overexpression of *Fgf6* in mouse skeletal muscle showed enhanced protein synthesis and muscle hypertrophy, which may be explained by the activation of the mTOR signaling pathway. Further, overexpression of *Fgf6* in the skeletal muscle not only provided partial protection against HFD-induced obesity but also improved glucose and lipid metabolism. To the best of our knowledge, this is the first report that links *Fgf6* expression in skeletal muscles to metabolic disorders, providing new insight into the role the environment-gene interaction network plays in the pathogenesis of obesity, and this insight might aid in the development of prevention and treatment strategies for metabolic diseases.

Genome-wide methylation and its association with obesity and T2DM, as well as with related phenotypes in the human skeletal muscle, have been investigated in recent years (17, 39–42). However, nearly all the studies are based on arrays or candidate genes, and thus the methylation sites covered are just the tip of the iceberg. In this study, we used MeDIP-Seq, a sequencing-based method with higher coverage of the human epigenome, and RNA-Seq, which resulted in the identification of *FGF6*, one of the many paracrine FGFs, as a modulator of metabolic homeostasis of skeletal muscles. *Fgf6*-knockout mice have been generated in previous studies to investigate the function of *Fgf6* on skeletal muscle regeneration, but the results have been inconsistent (43–46). Despite the contradictory conclusions in *Fgf6*-knockout mice, the importance of *Fgf6* in the maintenance of muscle stem cells, muscle cell differentiation, and related phenotypes is undeniable (47). In recent years, a novel link between endocrine FGFs and the skeletal muscle has been reported. Chronic exercise stimulated FGF23 production and was demonstrated to control excess reactive oxygen species production and enhance mitochondrial function in the skeletal muscle, enhancing physical performance during exercise (48). In addition, FGF19 and FGF21 have been reported to have opposite effects on skeletal muscle mass in mice (37, 49). Together with these reports, our study of *Fgf6* expands the understanding of the roles FGFs play in the skeletal muscle, especially in the regulation of metabolism.

Based on our sequencing analysis, we confirmed the altered expression of *FGF6* in the skeletal muscles of individuals with obesity in a larger cohort consisting of 35 pairs of human skeletal muscle specimens. Further, we showed that decreased expression of *FGF6* was mediated by hypermethylation in the promoter region, inhibiting the binding of CREB1. Previous studies have established the key role of CREB1 in the regulation of skeletal muscle mass. *Creb1*-null-mutant mice have been reported to exhibit strong myogenic defects (50), and mice expressing a dominant-negative *Creb1* transgene show muscle degeneration due to a reduced expression of *Sik1* (51). In addition, activated by β_2_-adrenergic agonists such as clenbuterol, CREB1 has been demonstrated to be an intermediate link, regulated by cAMP–protein kinase A, in targeting genes, such as *Ppargc1a*, *Nr4a3*, and *Kdm3a*, limiting muscle waste or increasing muscle fiber size (52, 53). We have therefore not only elucidated a potentially new mediator between CREB1 and skeletal muscle hypertrophy but also provided a potentially new target to treat muscle waste and metabolic disorders using technologies designed to manipulate DNA methylation at specific genomic loci (54).

A recent study has shown that administration of FGF19 causes skeletal muscle hypertrophy, by stimulating the phosphorylation of ERK1/2 and P70S6K1, and ameliorates obesity-induced skeletal muscle atrophy in mice (37). Herein, we found that overexpression of *Fgf6* in the skeletal muscle in mice caused a stark increase in muscle fiber size and strength and inhibited the expression of myostatin, a negative regulator of muscle mass. However, muscle mass did not change 1 month after *Fgf6* overexpression but increased 12 weeks later, with the HFD-FGF6 mice showing more remarkable increase of muscle mass than that in NCD-fed mice. Numerous textbooks used in the training of nutrition professionals advocate the creation of an energy surplus when attempting to facilitate skeletal muscle hypertrophy, emphasizing the importance of energy supply (55). Therefore, we considered that energy supply as well as duration of *Fgf6* overexpression may account for the discrepancy of changes in muscle mass. Similar to FGF19 administration, *Fgf6* overexpression led to an upregulation of p-ERK1/2^Thr202/Tyr204^ and p-P70S6K^Ser434^. It is worth noting that the expression of both the total and phosphorylated proteins relevant to the mTOR pathway increased with the overexpression of *Fgf6*, with long-term (12 weeks) overexpression of *Fgf6* increasing the protein expression and activity to a similar extent, except for that of P70S6K. This phenotype has also been reported in another study, which demonstrated that FST288 has the ability to induce muscle hypertrophy (32, 33). Apart from its impact on muscle mass, FST gene therapy has been proved to prevent HFD-induced obesity and completely normalize muscle glucose uptake in diet-induced insulin-resistant obese mice. In our study, the HFD-FGF6 mice exhibited an improved metabolism, reduced subcutaneous and visceral fat, relieved insulin resistance, elevated O_2_ consumption, and lower serum TG and NEFA levels, in comparison with those in the HFD-Ctrl group. Off-target effects of AAV9 were ruled out by detecting the expression of GFP, and the amelioration of insulin resistance in HFD-fed mice was only observed in the skeletal muscle of mice treated with AAV9-FGF6, but not in iWAT, eWAT, or liver. In addition, FGF6 also stimulates the phosphorylation of AMPK, which has been shown to promote glucose disposal and glucose lowering (56) by an increase in the AMP or ADP relative to ATP. Taken together, these findings confirm the role of the skeletal muscle in the improvement of metabolism after *Fgf6* overexpression. Nevertheless, despite the amelioration of HFD-induced metabolic phenotype presented here, we also observed increased fat deposition in skeletal muscles treated with *Fgf6*, especially in the HFD-fed mice, in the absence of prominent inflammation. Ectopic accumulation of adipose in skeletal muscle is usually associated with muscle disorders, aging, and metabolic diseases. However, considering the generation of intramuscular adipose tissue in the early stages of muscle regeneration in several models (57–60), fat deposition can also be recognized as a physiological process required for muscle regeneration (61). Furthermore, ablation of the adipogenic cell lineage or PPARγ, which is a lipid-activated transcription factor, impairs myogenesis after injury, highlighting the unappreciated role of the intermuscular adipose tissue in skeletal muscle regeneration (62, 63). We thus hypothesize that a functional adipogenic response is required to efficiently sustain myogenesis after *Fgf6* overexpression, without resulting in the development of inflammation or insulin resistance. Future studies are required to investigate the impact of *Fgf6* on intermuscular fat.

There are several limitations of our study. First, no detection of GFP in adipose tissues and liver does address the concern over potential viral spillover but does not directly address the possibility that overexpression of *Fgf6* in the muscle may, in and of itself, impact *Fgf6* expression in other tissues. Second, the effects of *Fgf6* on skeletal muscle hypertrophy and metabolic improvement were only demonstrated by AAV-mediated overexpression, and *Fgf6*-knockout mice exhibit inconsistent phenotypes of skeletal muscle regeneration (43–46); therefore, future studies focusing on skeletal muscle mass and whole-body metabolism of *Fgf6*-knockout mice fed with an HFD may help us to further understand the role played by *Fgf6*. Third, it is critical to conduct mouse models, with ablation of the adipogenic cell lineage or PPARγ, and investigate the crosstalk between muscle stem cells and fibrogenic or adipogenic progenitors to better characterize the interaction between adipogenesis and myogenesis in the muscles of *Fgf6*-treated mice.

In conclusion, our findings uncover a potentially new function of *Fgf6* in the regulation of skeletal muscle protein synthesis and metabolic homeostasis. The development of strategies targeting *Fgf6*, including DNA methylation editing or AAV gene therapy, can be promising in regulating metabolism in patients with obesity.

## Methods

### Human specimens.

Muscle biopsies were obtained from the vastus lateralis muscle of patients who underwent orthopedic surgery during 2012–2014 at the Shanghai Jiao Tong University Affiliated Sixth People’s Hospital. All the samples included in our study were paired, matched by sex and age. Each pair contained 1 sample from participants with a normal body mass index and the other from individuals who were obese according to the criteria provided by the Ministry of Health of the People’s Republic of China (64). MeDIP-Seq and RNA-Seq were performed for 7 pairs of skeletal muscle specimens in the preliminary screening stage. Four genes that were differentially methylated in the promoter region and differentially expressed were further validated in a larger cohort of 35 pairs. The clinical characteristics of the participants are shown in Supplemental Tables 1 and 3.

### Animals.

Male C57BL/6J mice, aged 6 weeks, were purchased from the Nanjing Biomedical Research Institute of Nanjing University. Mice were housed at 21°C ± 1°C, with a humidity of 55% ± 10%, and under a 12-hour light/12-hour dark cycle. Mice were fed either an NCD or an HFD (60% kcal from fat, 20% kcal from carbohydrate and 20% kcal from protein; D12492, Research Diets) at the age of 8 weeks, after administration of AAV. The mice had ad libitum access to food and water during the course of the experiment.

### MeDIP-Seq.

Genomic DNA (gDNA) was extracted from the human skeletal muscle specimens using the QIAamp DNA Mini Kit (QIAGEN) according to the manufacturer’s protocol. The gDNA samples were electrophoresed on agarose gels, and the optical density ratios were measured to confirm the integrity and purity and to estimate the concentration of the extracted gDNA. The gDNA was subjected to fragmentation, and the methylated DNA fragments were isolated using the SimpleDIP Methylated DNA IP (MeDIP) Kit (Cell Signaling Technology). Next, DNA libraries for the isolated fragments were constructed using the TruSeq DNA LT Sample Prep Kit (Illumina) and subjected to single-read sequencing, with a 50 bp read length, using the Illumina HiSeq 2500 platform. The resulting raw sequencing data were assigned to different samples according to the barcodes, and FastQC was applied to estimate the read quality. Adapters and low-quality reads were removed by Trimmomatic software (version 0.36). The cleaned reads were aligned to the hg19 genome using the Burrows-Wheeler Alignment tool (version 0.7.15). The repeat sequences were marked using Picard-tools (version 2.5.0), and the R package MEDIPS was employed to calculate the saturation and coverage of the CpG islands. The genome was divided into continuous windows of 100 bp size by MEDIPS, and the reads corresponding to each window were counted and normalized. The differential methylation windows were calculated by the edgeR package in R. The script annotatePeaks.pl included in Homer was used to identify the genes or adjacent genes and the detailed annotated information for the differentially methylated regions. The promoter regions were identified as 1000 bp upstream and 100 bp downstream of the TSS (–1000 to +100 bp from the TSS). The threshold for significant differentially methylated regions was set at *P* < 0.05.

### RNA-Seq.

Total RNA was extracted from human and mouse skeletal muscle specimens using the TRIzol reagent (Invitrogen, Thermo Fisher Scientific). The quantity and quality of each mRNA sample were examined using NanoDrop (Thermo Fisher Scientific) and electrophoresis, respectively. The RNA libraries were constructed using the TruSeq RNA LT Sample Prep Kit v2 (Illumina) according to the manufacturer’s protocol, and paired-end reads were obtained on the Illumina HiSeq 2500 platform. Quality of the RNA-Seq data was estimated using RSeQC software (version 2.6.4), and the script geneBody_coverage.py was used to calculate the coverage of the reads over the gene body. Only the paired reads without adapter sequences, with an average Phred quality score ≥ 15 and read length ≥ 36, were retained for paired-end sequencing data. Then, the cleaned reads were aligned to the index file of the hg19 or mm10 genome using HISAT2 (version 2.0.5). The aligned Sequence Alignment/Map (SAM) files were converted to Binary Alignment Map files and sorted by read name using SAMtools (version 1.4). Read counts for each gene were generated using HTSeq-count; the read counts were normalized and the differentially expressed genes were determined using the edgeR package in R. The threshold of significantly differentially expressed genes was set at *P* < 0.05 to compare results of the RNA-Seq with MeDIP-Seq results. The Gene Expression Omnibus accession number is GSE182686.

### DNA isolation, bisulfite conversion, and DNA methylation analysis.

The gDNA (500 ng) was extracted from skeletal muscle specimens with the QIAamp DNA Mini Kit (QIAGEN) according to the manufacturer’s instructions. Bisulfite conversion was then performed using the EpiTect Fast DNA Bisulfite kit (QIAGEN). PyroMark Assay Design SW 2.0 (QIAGEN) was used to design the amplification and sequencing primers (Supplemental Table 7), and the methylation level of each CpG site was measured by pyrosequencing using PyroMark Q24 machine (QIAGEN).

### RNA isolation and quantitative PCR.

RNA from human and mouse tissue specimens was extracted using the RNeasy Plus Universal Kit (QIAGEN). The mRNA was reverse-transcribed into cDNA using the PrimeScript RT reagent kit with the gDNA Eraser (Takara). qPCR assays were performed on the Applied Biosystems 7900HT Fast Real-Time PCR System using TaqMan fluorescence probe (Applied Biosystems, Thermo Fisher Scientific) or SYBR Green Premix Ex Taq (Roche). Relative mRNA expression was quantified after normalizing against the expression of housekeeping genes *PPIA* or *Gapdh*. The assay ID as well as sequences of primers are provided in Supplemental Table 8.

### Plasmids, transfections, and luciferase reporter assays.

JASPAR (http://jaspar.genereg.net/) was used to analyze the potential transcription factor binding sites in the promoter of *FGF6* (65). The WT *Fgf6* promoters were amplified from mouse gDNA and subcloned into the pGL3-basic luciferase reporter vector with *Kpn*I or *Bgl*II restriction sites. The mutant *Fgf6*-luciferase reporter with a deletion at the putative CREB1 or MYC binding site (CRE or E-box) was generated using the QuikChange II Site-Directed Mutagenesis Kit (Agilent). Primers are listed in Supplemental Table 8. The CREB1 and MYC expression plasmids were purchased from Bioworld Technology. For the luciferase reporter assays, 100 ng WT or mutant *Fgf6* reporter vector, 200 ng CREB1 or MYC expression plasmid, and 10 ng pRL-TK (renilla luciferase control reporter vector) were cotransfected into HEK293T cells (ATCC) in duplicate 24-well plates using Lipofectamine 2000 (Invitrogen, Thermo Fisher Scientific), according to the manufacturer’s instructions. Twelve hours after transfection, the medium was replaced with fresh high-glucose Dulbecco’s modified Eagle’s medium (DMEM) containing 10% fetal bovine serum (FBS), with 10 μM forskolin (MilliporeSigma), 5 μM 5-Aza (MilliporeSigma), or DMSO (vehicle), and incubated for another 36 hours. Cells were harvested to analyze the relative luciferase activity using dual-luciferase reporter assay system (Promega).

### ChIP-qPCR assay.

ChIP assays were performed using the SimpleChIP Enzymatic Chromatin IP Kit (9003; Cell Signaling Technology) according to the manufacturer’s protocol. Briefly, gastroc tissue specimens obtained from 8-week-old mice were minced into small pieces and cross-linked with 1% formaldehyde at room temperature for 10 minutes before quenching with a 0.125 M glycine solution. Tissue pieces were then resuspended in ice-cold phosphate-buffered saline (PBS) and ground with a Dounce homogenizer. The cross-linked chromatin fragments were then sheared using a sonicator (Scientz-IID; Scientz) into 200–1000 bp long fragments. Then, 10 μg of anti–p-CREB (9198; Cell Signaling Technology), anti–c-MYC (18583; Cell Signaling Technology), or control IgG (3900; Cell Signaling Technology) antibodies were used for immunoprecipitation. The protein-DNA cross-links were reversed overnight at 65°C, and the DNA fragments were purified and diluted for qPCR (details of the primers used are given in Supplemental Table 8).

### Production and delivery of the AAV vector.

AAV9-containing plasmids with a skeletal muscle–specific creatine kinase promoter (d*MCK*, a gift from Qiurong Ding, Shanghai Institutes for Biological Sciences, Chinese Academy of Sciences, Shanghai, China) (27) were generated for the overexpression of *Fgf6* in the mouse skeletal muscle (AAV9-FGF6; Figure 2A). A viral vector without an *Fgf6* cDNA insertion was used as the control (AAV9-Ctrl; Figure 2A). AAV injection was performed in 8-week-old mice, which were divided into 2 cohorts based on different modes of AAV administration. The first cohort was the self-comparison cohort, wherein 50 μL of 10^11^ vector genomes (vg) of AAV9-FGF6 and AAV9-Ctrl were injected into either side of the gastroc muscle (Figure 2B). These mice were fed with an NCD and sacrificed 1 month later. The second was the unique expression cohort, wherein 10^11^ vg of AAV9-FGF6 or AAV9-Ctrl was directly injected into bilateral quads, gastroc, and TA muscles of mice, resulting in *Fgf6* overexpression or control mice, respectively. Mice in the second cohort were randomly assigned to the NCD or HFD group for a total of 12 weeks.

### Grip strength test.

Muscle strength of mice was measured by a digital grip strength meter (Yiyan Technology). Mice were allowed to hold on to a metal pull bar with 4 paws or forelimbs and were gently pulled backward by the tail until the animals could no longer hold the pull bar. The force at the time of release was recorded as the peak tension. Each mouse was given 5 trials with 4 paws and 8 trials with forelimbs. Grip strength of hind limbs, which were treated with AAV9-Ctrl or AAV9-FGF6, was calculated by subtracting the grip strength of forelimbs from that of all limbs. The investigator was blinded to the animal group treatment.

### GTT and ITT.

The GTT and ITT were performed in the mice of the second cohort 10 and 11 weeks after AAV delivery, respectively. For the GTT, mice were fasted overnight (16 hours) and intraperitoneally injected with D-(+)-glucose (MilliporeSigma) solution at a dose of 1.2 g/kg body weight. For the ITT, mice were fasted for 4 hours and intraperitoneally injected with insulin (0.75 U/kg body weight for the NCD group and 1.25 U/kg body weight for the HFD group; Novo Nordisk). Blood glucose levels were measured in the tail vein blood before and 15, 30, 60, 90, and 120 minutes after glucose or insulin injection.

### Body composition analysis and metabolic cage analysis.

Fat mass and lean mass were assessed using a whole-body composition analyzer (EchoMRI) according to the manufacturer’s instructions. The comprehensive laboratory animal monitoring system (Columbus Instruments) was used to monitor the physical activity and metabolism. Mice were placed individually in a chamber for 72 hours, wherein the first 24 hours served as an adaptive phase, and the oxygen consumption (VO_2_), food intake, and total movement were recorded for analysis in the following 48 hours.

### Tissue collection.

Mice were anesthetized with 1% pentobarbital, and blood from orbital plexus was collected. Blood samples were allowed to clot for 2 hours at room temperature before centrifugation for 20 minutes at 2000*g*. Serum was isolated and stored at –80°C for subsequent biochemical testing. The skeletal muscles, consisting of quads, gastroc, TA, and soleus; adipose tissues, consisting of iWAT, eWAT, and BAT; and liver of each mouse were dissected, weighed, and collected for further analysis. For the detection of insulin signaling, mice were intraperitoneally injected with insulin (1 U/kg, Novo Nordisk), and tissues were harvested 15 minutes after injection.

### Measurement of fiber size.

Skeletal muscle samples were fixed in 10% formalin, paraffin-embedded, and cut into 4 μm transverse sections. Sections were stained with H&E, and images were acquired on a Leica DM4 B microscope. One month after AAV injection, the CSA of 3004 fibers in AAV9-Ctrl mice and 2963 fibers in AAV9-FGF6 mice were calculated using Image-Pro Plus software (version 6.0). In addition, 5515 fibers in HFD-Ctrl mice and 5712 fibers in HFD-FGF6 mice were included for fiber size analysis.

### TG quantification for skeletal muscle.

TGs in skeletal muscle were quantified after extraction (66). Briefly, 30–50 mg of mouse skeletal muscle was homogenized in 500 μL of ice-cold PBS and then centrifuged at 12,000*g* at 4°C for 10 minutes. Next, 50 μL of the upper phase was taken to determine protein concentrations. To extract TGs, a 2:1 (v/v) mixture of chloroform/methanol (1.5 mL) was added to each sample by vortexing. Samples were then centrifuged at 2000*g* at 4°C for 10 minutes. The organic (lower) phase was air-dried in a fresh tube and then resuspended in 600 μL of 1% Triton X-100 in ethanol. TGs were measured using kits from Siemens Healthcare Diagnostics Inc. on the ADVIA 2400 Chemistry System.

### Estimation of protein synthesis.

Measurements of protein synthesis levels were performed using the SUnSET technique (31, 67). Briefly, mice received an intraperitoneal injection of 0.04 μmoL/g body weight puromycin (MilliporeSigma), dissolved in 100 μL of PBS. Thirty minutes after the injection, muscle samples were collected and frozen in liquid nitrogen. Puromycin levels were analyzed by Western blotting.

### Western blotting.

Tissues were extracted using radioimmunoprecipitation lysis buffer (Beyotime), containing 50 mM Tris (pH 7.4), 150 mM NaCl, 1% Triton X-100, 1% sodium deoxycholate, 0.1% sodium dodecyl sulfate (SDS), sodium orthovanadate, sodium fluoride, EDTA, and leupeptin, with protease and phosphatase inhibitors (Roche). Concentrations of protein samples were determined using the BCA protein assay kit (Beyotime). Equivalent amounts of protein samples were separated using SDS-polyacrylamide gel electrophoresis and then wet-transferred onto nitrocellulose membranes (MilliporeSigma). After blocking with 5% skim milk (Cell Signaling Technology) for 1 hour at room temperature, membranes were incubated with primary antibodies overnight at 4°C, followed by incubation with horseradish peroxidase–conjugated secondary antibody (anti–rabbit IgG, HRP-linked antibody, 7074, Cell Signaling Technology, or anti–mouse IgG, HRP-linked antibody, 7076, Cell Signaling Technology) for 1 hour at room temperature. Protein bands were visualized using the ECL Chemiluminescent Kit (MilliporeSigma) on the ChemiDoc Touch Imaging System (Bio-Rad). The primary antibodies used are listed in Supplemental Table 9.

### Mouse serum lipid and GDF-8/myostatin analysis.

Serum levels of cholesterol and TG were measured using kits from Siemens Healthcare Diagnostics Inc. on the ADVIA 2400 Chemistry System. The NEFA levels were determined by an enzymatic endpoint method (NEFA FS; DiaSys Diagnostic Systems). A sandwich enzyme-linked immunosorbent assay kit (GDF-8/Myostatin Immunoassay, DGDF80; R&D Systems, Bio-Techne) was used to determine serum myostatin concentrations.

### Transmission electron microscopy for skeletal muscle mitochondria density.

Excised gastroc muscles were sliced at 2 mm × 2 mm × 2 mm cubes, fixed in 2.5% glutaraldehyde (Servicebio) at 4°C for 2–4 hours, and postfixed in 1% osmium tetroxide in 0.1 M sodium cacodylate buffer for 2 hours at room temperature. Samples were then dehydrated in a series of ethanol (15 minutes each; 50%, 70%, 95%, twice at 100%) and twice with acetone (15 minutes each). After resin penetration and polymerization, ultrathin sections (60–80 nm) were taken on the Ultra microtome (UC7, Leica) and poststained with uranyl acetate and lead stain. Samples were observed using transmission electron microscopy (HT7700, hitachi) and images were acquired. For quantification of mitochondria, 45 images per group were analyzed.

### Seahorse biosystems.

The C2C12 cells (mouse skeletal myoblasts) were obtained from ATCC and cultured in DMEM containing 4.5 g/L glucose with 10% FBS. When cell confluence reached 80%, the medium was switched to differentiation medium (DMEM containing 3% horse serum). Real-time measurement of the oxygen consumption rate (OCR) was carried out using the XF24 Extracellular Flux Analyzer (Agilent) 24 hours after adding PBS (vehicle) or recombinant FGF6 protein (100 ng/mL) to day 4 differentiation myotubes. The OCR measurements were made before and after the sequential injection of 1 μM oligomycin, 1.5 μM FCCP, and 2 μM rotenone/antimycin A. The OCR levels were expressed with or without normalizing to total protein content.

### AMP/ATP and ADP/ATP levels.

The frozen gastroc muscle (20–30 mg) was ground in 700 μL of methanol. The lysate was then mixed with 700 μL of chloroform and 350 μL of water by 20 seconds of vortexing. After centrifugation at 13,500*g* for 20 minutes at 4°C, 700 μL of the aqueous phase was collected and freeze-dried in a vacuum concentrator, then dissolved in 50 μL water and 50 μL acetonitrile. Finally, 80 μL of redissolved solution was loaded for analysis after centrifugation at 13,500*g* for 20 minutes at 4°C. The levels of AMP, ADP, and ATP were measured using ultra-performance liquid chromatography–tandem mass spectrometry (ACQUITY, Waters), and the MassLynx software (version 4.1) was used for data processing.

### Statistics.

Statistical analysis was performed using the SAS software (version 9.4) and GraphPad Prism 7 (GraphPad Software). Results are represented as mean ± standard error of mean or median (interquartile range, 25%–75%) after testing for normality using the Shapiro-Wilk test. Two-tailed paired or unpaired Student’s *t* test and Wilcoxon’s signed rank sum test were performed to compare 2 groups when appropriate. One-way ANOVA correcting for multiple comparisons by controlling the FDR was used when comparing more than 2 groups. A 2-tailed *P* value of less than 0.05 was considered statistically significant.

### Study approval.

Experiments involving humans were approved by the Human Research Ethics Committee of Shanghai Jiao Tong University Affiliated Sixth People’s Hospital, and written informed consent was obtained from all participants. All animal experiments were performed according to procedures approved by the Animal Care Committee of Shanghai Jiao Tong University Affiliated Sixth People’s Hospital.

## Author contributions

WJ and CH conceived and designed the research. BX, CL, and RZ performed the experiments and collected and analyzed data. MT, YH, and LJ assisted with experiments. HZ analyzed the omics data. BX drafted the manuscript. LX contributed many suggestions and helpful discussions on the experiments and manuscript. All authors were involved in writing the paper and had final approval of the submitted and published versions. The order of the co–first authors in the author list was decided as follows: BX was involved in the study from the initial stages and CL helped complete it.

## Supplementary Material

Supplemental data

## Version 1 09/07/2021

In-Press Preview

## Version 2 10/08/2021

Electronic publication
