# Supplementary material for: Skeletal muscle–targeted delivery of Fgf6 protects mice from diet-induced obesity and insulin resistance
Source: JCI Insight. 2021 Oct 8;6(19):e149969. doi: 10.1172/jci.insight.149969 (PMC8525645; doi:10.1172/jci.insight.149969)
Supplement: Supplemental data [file jciinsight-6-149969-s058.pdf]

Supplementary Figures

Supplemental Figure 1. Methylome profiling of skeletal muscle specimens from normal-weight individuals and participants with obesity.

Supplemental Figure 2. Transcriptome profiling of skeletal muscle specimens from normal-weight individuals and participants with obesity.

Supplemental Figure 3. Validation of differentially expressed genes in skeletal muscle of human with obesity and experiments exploring transcriptional regulation of *FGF6*.

Supplemental Figure 4. The effect of *Fgf6* overexpression on skeletal muscle.

Supplemental Figure 5. Overexpression of *Fgf6* in skeletal muscle improves glycometabolism of mice fed on a normal chow diet (NCD) but does not affect insulin sensitivity of adipose tissues and liver in high-fat diet (HFD)-fed mice.

Supplemental Figure 6. FGF6 increases the basal oxygen consumption and activats AMP-activated protein kinase (AMPK) in muscle cells.

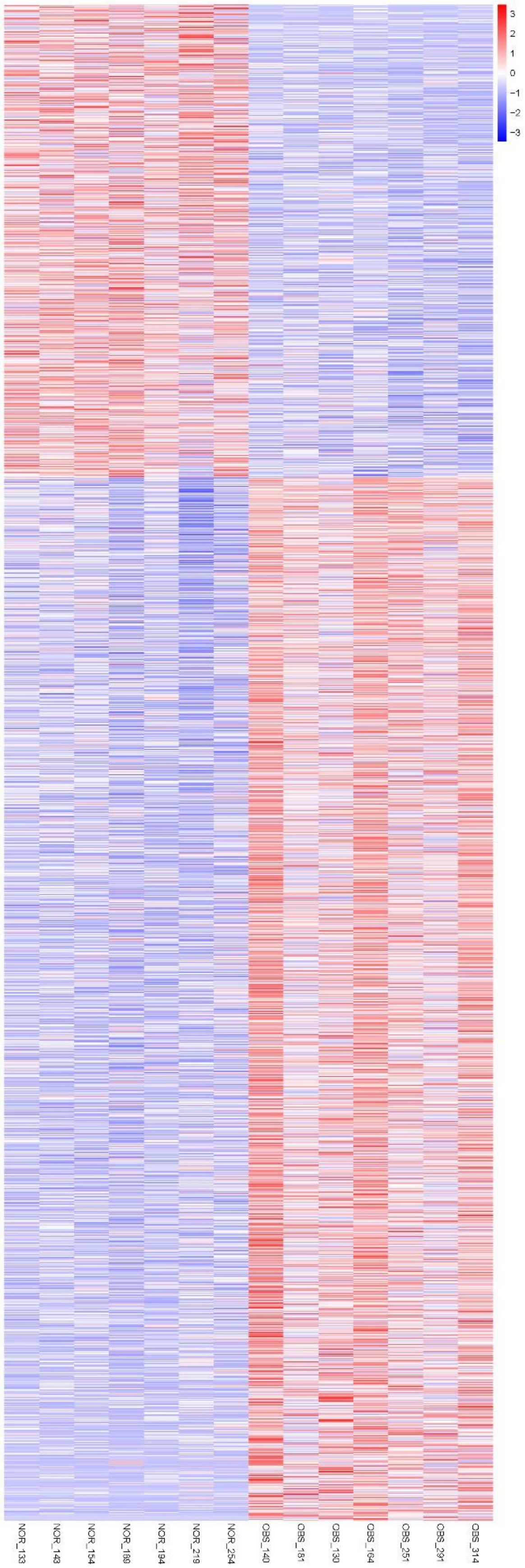

Supplemental Figure 1.

Supplemental Figure 1. Methylome profiling of skeletal muscle specimens from normal-weight individuals and participants with obesity. Heatmap of hypermethylated and hypomethylated fragments (2249 genes containing 4080 fragments, $P < 0.05$) in skeletal muscle specimens from participants with obesity (OBS; n = 7) compared with that in skeletal muscle specimens from normal-weight individuals (NOR; n = 7).

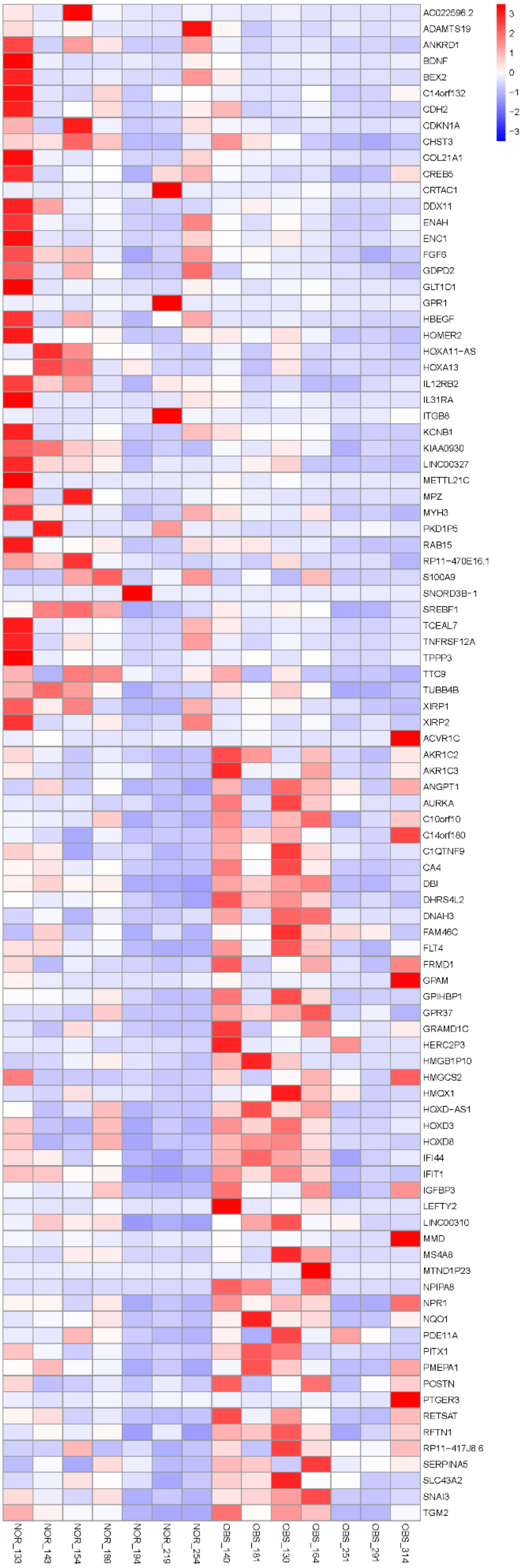

Supplemental Figure 2.

Supplemental Figure 2. Transcriptome profiling of skeletal muscle specimens from normal-weight individuals and participants with obesity. Heatmap of upregulated and downregulated genes (94 DEGs, $P < 0.05$) in skeletal muscle specimens from individuals with obesity (OBS; n = 7) compared with that in skeletal muscle specimens from normal-weight individuals (NOR; n = 7).

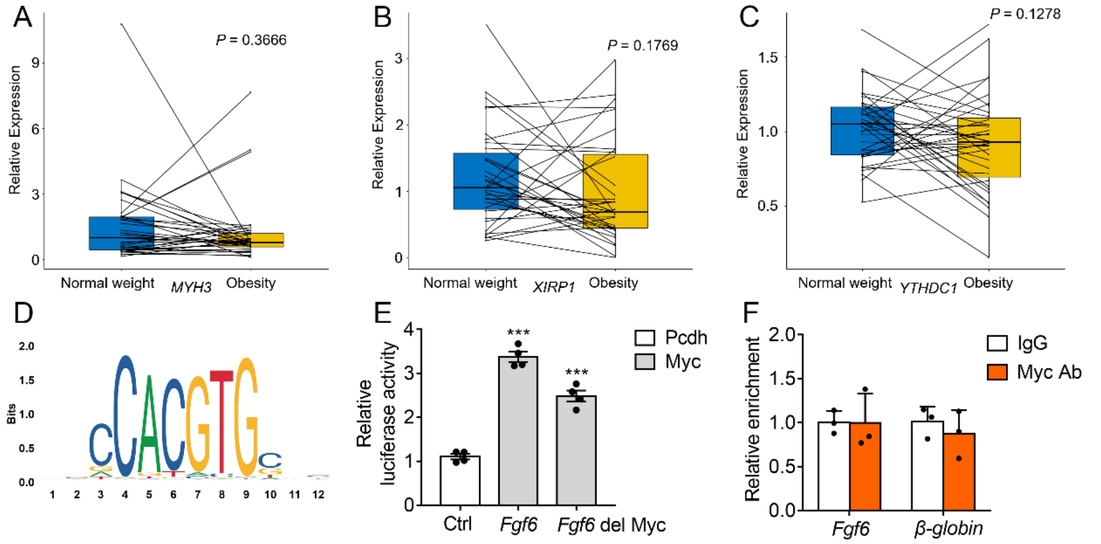

Supplemental Figure 3.

Supplemental Figure 3. Validation of differentially expressed genes in skeletal muscle of human with obesity and experiments exploring transcriptional regulation of *FGF6*. (**A**–**C**) Comparison of relative mRNA levels of *MYH3* (**A**), *XIRP1* (**B**), and *YTHDC1* (**C**) estimated by quantitative real time polymerase chain reaction (qPCR) in 35 pairs of age-matched skeletal muscle specimens from normal-weight individuals and participants with obesity (19 male pairs and 16 female pairs). (**D**) Enhancer box near the transcriptional start site of *FGF6*. (**E**) Luciferase activities of HEK-293T cells transfected with wild type *Fgf6* luciferase reporter construct or mutant *Fgf6* luciferase reporter, wherein the promoter lacks the MYC binding site (*Fgf6* del Myc), and a plasmid overexpressing MYC or an empty vector (n = 4). (**F**) Chromatin immunoprecipitation-qPCR analysis of MYC enrichment in the *Fgf6* promoter in skeletal muscles of mice (n = 3). *β-globin* was used as a negative control.

Data information: The box plot represents data from the first quartile to the third quartile. The second quartile represents the median of the data (**A**–**C**). Other results are represented as the mean ± standard error of mean (**E** and **F**). Statistical analysis was performed using paired and unpaired Student's *t*-tests or Wilcoxon signed rank sum tests where appropriate. *$P < 0.05$, **$P < 0.01$, ***$P < 0.001$.

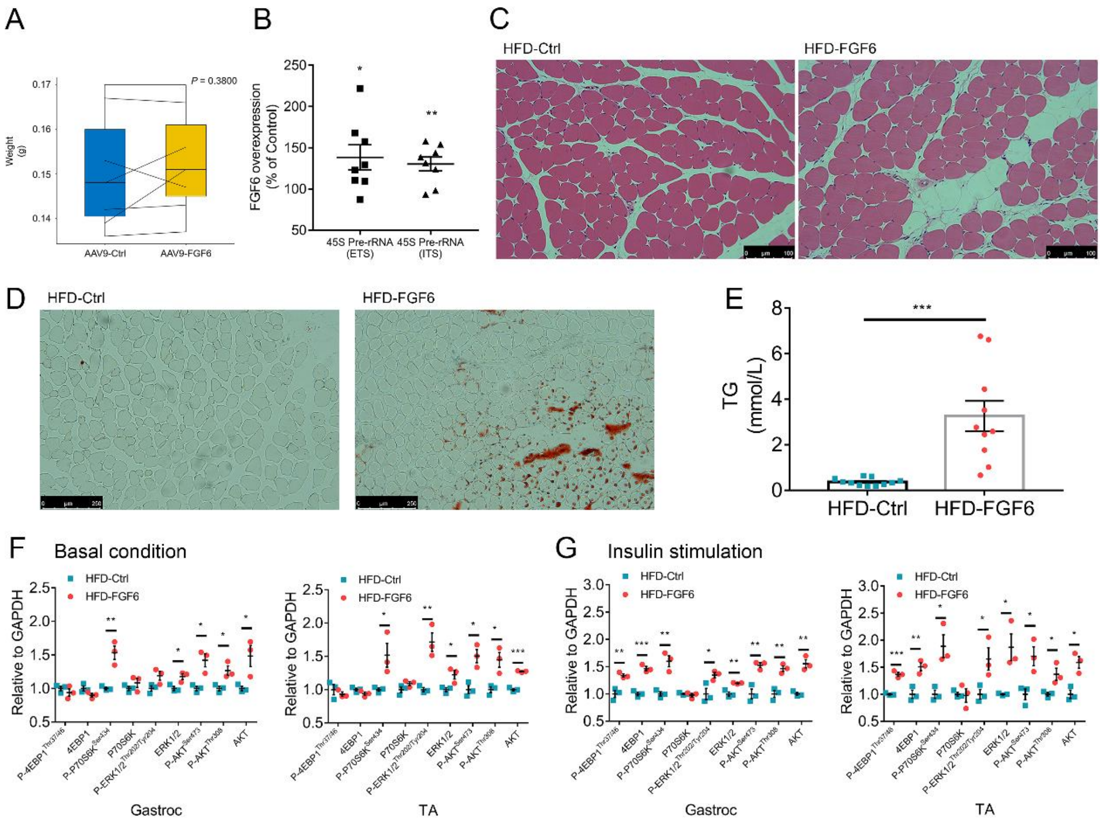

Supplemental Figure 4.

Supplemental Figure 4. The effect of *Fgf6* overexpression on skeletal muscle. (**A**) Measurements of gastrocnemius (gastroc) muscle weight in mice one month after injection of adeno-associated virus (AAV) 9-control (Ctrl) or AAV9-FGF6 (n = 7). (**B**) Comparison of relative mRNA levels of 45S Pre-rRNA, estimated by qPCR, in muscles one month after AAV9-Ctrl or AAV9-FGF6 injection (n = 8). (**C** and **D**) Images of hematoxylin and eosin-stained (**C**) and Oil Red O-stained (**D**) sections of gastroc muscles in high-fat diet (HFD)-fed mice. Scale bars, 100 µm. (**E**) Analysis of triglyceride content in the gastroc muscles of HFD-fed mice injected with AAV9-Ctrl (n = 11) or AAV9-FGF6 (n = 10). (**F** and **G**) The integrated density of the phosphorylated and total protein band in lysates of gastroc (Figure **5J**) and tibialis anterior (TA; Figure **5K**) muscles, in basal (**F**) or insulin-stimulated (**G**) conditions, was quantified (n = 3 per group).

Data information: The box plot represents data from the first quartile to the third quartile. The second quartile represents the median of the data (**A**). Other results are represented as the mean ±standard error of mean (**B**, **E**, **F**, and **G**). Statistical analysis was performed using paired and unpaired Student's *t*-tests where appropriate. *$P < 0.05$, **$P < 0.01$, ***$P < 0.001$.

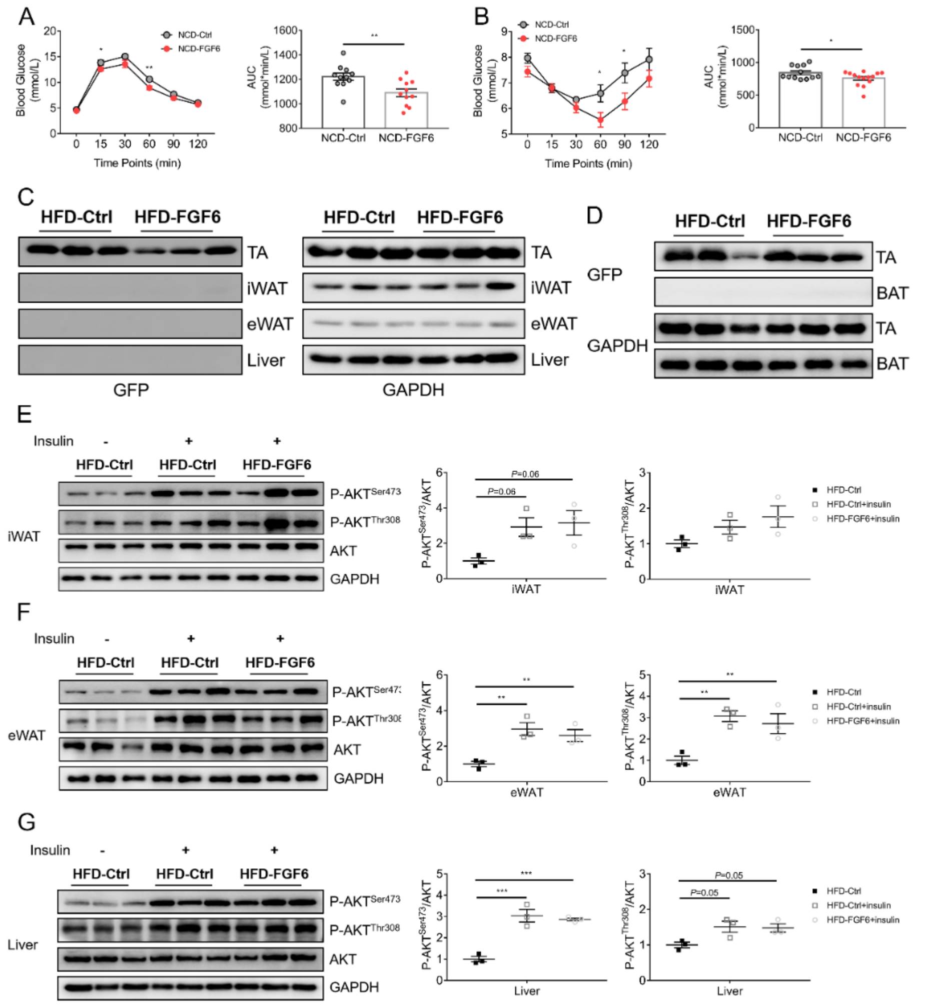

Supplemental Figure 5.

Supplemental Figure 5. Overexpression of *Fgf6* in skeletal muscle improves glycometabolism of mice fed on a normal chow diet (NCD) but does not affect insulin sensitivity of adipose tissues and liver in high-fat diet (HFD)-fed mice. (**A** and **B**) Glucose tolerance test (**A**) and insulin tolerance test (**B**) in NCD-fed mice at 10 weeks and 11 weeks, respectively (n = 11–14 per group). AUC, area under the curve. (**C**) Detection of GFP protein in lysates (30 µg of proteins from each sample) from tibialis anterior (TA) muscle, inguinal subcutaneous white adipose tissue (iWAT), epidydimal white adipose tissue (eWAT), and liver (n = 3 per group). (**D**) Detection of GFP protein in lysates (30 µg of proteins from each sample) from TA muscle and brown adipose tissue (BAT; n = 3 per group). (**E**–**G**) Quantification of P-AKT^{Ser473}/AKT ratio and P-AKT^{Thr308}/AKT ratio in iWAT (**E**), eWAT (**F**), and liver (**G**) in response to insulin stimulation in HFD-control (Ctrl) and HFD-FGF6 mice (n = 3 per group).

Data information: Results are represented as the mean ± standard error of mean. Statistical analysis was performed using unpaired Student's *t*-tests or One-way ANOVA correcting for multiple comparisons by controlling the False Discovery Rate where appropriate. **P* < 0.05, ***P* < 0.01, ****P* < 0.001.

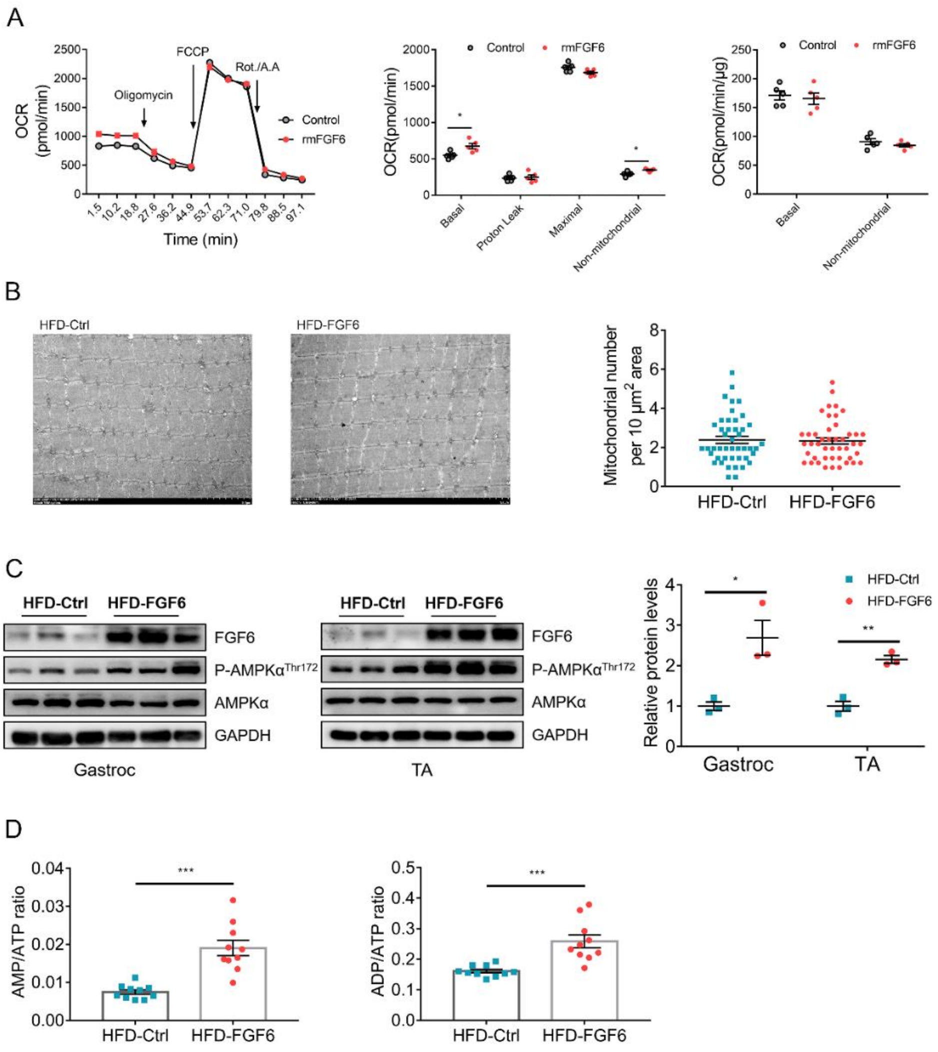

Supplemental Figure 6.

Supplemental Figure 6. FGF6 increases the basal oxygen consumption and activats AMP-activated protein kinase (AMPK) in muscle cells. (**A**) C2C12 myotubes were treated with medium containing either PBS (vehicle) or recombinant FGF6 protein (100 ng/mL) for 24 hours and then subjected to Seahorse bioanalyzer system to assess oxygen consumption rate (OCR; n = 5 per group). (**B**) Mitochondrial density of gastrocnemius (gastroc) musles from high-fat diet (HFD)-control (Ctrl) and HFD-FGF6 mice, assayed by transmission electron micrographs. Scale bars, 5 μm. 45 images per group were analyzed. (**C**) Quantification of P-AMPKα^{Thr172}/AMPK ratio in gastroc and tibialis anterior (TA) muscles in HFD-Ctrl and HFD-FGF6 mice (n = 3 per group). (**D**) AMP/ATP and ADP/ATP ratios in gastroc muscles from HFD-Ctrl and HFD-FGF6 mice, determined by ultra-performance liquid chromatography-tandem mass spectrometry (n = 10 per group).

Data information: Results are represented as the mean ± standard error of mean. Statistical analysis was performed using unpaired Student's *t*-tests. $*P < 0.05$, $**P < 0.01$, $***P < 0.001$.

Supplementary Tables

Supplemental Table 1. Clinical characteristics of participants in the preliminary screening stage

Supplemental Table 2. Genes hypermethylated in the promoter region but downregulated in the skeletal muscle of participants with obeisty

Supplemental Table 3. Clinical characteristics of participants in the validation stage

Supplemental Table 4. DNA methylation level of *FGF6* promoter region in human skeletal muscle

Supplemental Table 5. Difference of DNA methylation level of *FGF6* promoter region between skeletal muscle samples from participants with obesity and normal-weight individuals

Supplemental Table 6. Promoter scanning results from JASPAR Database

Supplemental Table 7. Primers for DNA methylation analysis

Supplemental Table 8. Assay ID, and primer sequence for quantitative PCR and plasmids construction

Supplemental Table 9. Primary antibodies for Western blots

**Supplemental Table 1. Clinical characteristics of participants in the preliminary screening stage**				
	Normal weight	Obesity	Obesity–Normal weight	*P*–Value
N	7	7	7	/
Sex (Male / Female)	7 / 0	7 / 0	/	/
Age (years)	48.29 ± 6.15	48.71 ± 6.15	0.43 ± 1.00	0.6822
BMI (kg/m^2)	21.57 ± 0.42	29.51 ± 0.90	7.94 ± 1.10	0.0004
Data are shown as mean ± standard error of mean.				

**Supplemental Table 2. Genes hypermethylated in the promoter region but downregulated in the skeletal muscle of participants with obesity**	
Genes	Full Name
MYH3	myosin heavy chain 3
FGF6	fibroblast growth factor 6
YTHDC1	YTH domain containing 1
MYH6	myosin heavy chain 6
CMTM5	CKLF like MARVEL transmembrane domain containing 5
CYP26B1	cytochrome P450 family 26 subfamily B member 1
SLC47A2	solute carrier family 47 member 2
ADAMTS19	ADAM metallopeptidase with thrombospondin type 1 motif 19
XIRP1	xin actin binding repeat containing 1
FAM135B	family with sequence similarity 135 member B
SHISA3	shisa family member 3
SLC26A9	solute carrier family 26 member 9
NEU4	neuraminidase 4

Supplemental Table 3. Clinical characteristics of participants in the validation stage				
	Normal weight	Obesity	Obesity–Normal weight	*P*–Value
N	35	35	35	/
Sex (Male / Female)	19 / 16	19 / 16	/	/
Age (years)	54.40 ± 2.34	54.37 ± 2.35	0 (0-0)	0.9271
BMI (kg/m^2)	21.21 ± 0.24	29.38 (28.41–32.05)	8.61 (6.56–11.47)	<.0001
SBP, mm Hg	123.77 ± 2.62	132.69 ± 3.43	8.91 ± 3.66	0.0202
DBP, mm Hg	74.43 ± 1.23	78 (70–84)	3.26 ± 2.85	0.2609
TG, mmol/L	1.28 (0.90–1.77)	1.81 (1.30–2.41)	0.94 ± 0.35	0.0184
TC, mmol/L	4.82 ± 0.24	4.67 ± 0.20	0.02 ± 0.31	0.9499
LDL-c, mmol/L	2.85 ± 0.17	2.81 ± 0.17	-0.14 ± 0.30	0.661
HDL-c, mmol/L	1.22 ± 0.06	1.10 ± 0.05	-0.11 ± 0.10	0.2817
FPG, mmol/L	5.06 ± 0.10	5.16 (4.85–6.14)	0.11 (-0.14–0.8)	0.0205
ALT, U/L	17 (13–27)	21 (15–26)	5 (-9–14)	0.2199
AST, U/L	20 (17–24)	21 (17–24)	2 (-5–8)	0.6645
ALP, U/L	73 (65–89)	73.97 ± 2.76	-2 (-21–7)	0.2608
GGT, U/L	24 (17–37)	35 (22–51)	9 (-2–27)	0.0619
ALB, g/L	45.94 ± 0.63	45 (44–48)	-0.54 ± 0.78	0.4902
CR, µmol/L	67 (58–78)	67.66 ± 2.49	-1 (-14–12)	0.7761
BUN, mmol/L	4.9 (4.0–5.8)	5.06 ± 0.19	0.2 (-0.8–1.3)	0.469
UA, mmol/L	298 (246–359)	369.63 ± 13.45	55.80 ± 17.60	0.0032
Abbreviations: BMI, body mass index; SBP, systolic blood pressure; DBP, diastolic blood pressure; TG, triglyceride; TC, total cholesterol; LDL-c, low-density lipoprotein cholesterol; HDL-c, high-density lipoprotein cholesterol; FPG, fasting plasma glucose; ALT, alanine aminotransferase; AST, aspartate aminotransferase; ALP, alkaline phosphatase; GGT: gamma-glutamyl transpeptidase; ALB, albumin; CR, creatinine; BUN, blood urea nitrogen; UA, uric acid. Data are shown as mean ± standard error of mean or median (interquartile range, 25–75%).				

Supplemental Table 4. DNA methylation level of *FGF6* promoter region in human skeletal muscle				
	CpG sites	Location (distance to TSS)	Mean value (%)	Level of methylation (%)
Region 1	CpG 1	–286	59.67	59.67 ± 1.32
	CpG 2	–278	47.64	45.59 (38.42–55.45)
	CpG 3	–270	64.03	64.03 ± 1.46
	CpG 4	–247	55.09	55.09 ± 1.42
Region 2	CpG 5	–192	68.71	68.71 ± 1.27
	CpG 6	–183	74.89	74.89 ± 1.17
	CpG 7	–179	67.94	67.94 ± 1.19
	CpG 8	–167	76.95	77.41 (74.29–82.22)
	CpG 9	–161	74.46	74.46 ± 0.94
	CpG 10	–144	76.80	76.98 ± 1.01
	CpG 11	–140	74.10	74.21 (70.32–79.54)
	CpG 12	–138	67.48	67.48 ± 0.82
	CpG 13	–107	75.63	75.63 ± 1.21
	CpG 14	–99	66.19	66.19 ± 1.30
Region 3	CpG 15	–38	61.06	59.55 (51.79–68.84)
	CpG 16	–30	61.84	60.54 (51.63–69.12)
	CpG 17	–7	63.18	63.18 ± 1.06
	CpG 18	23	43.58	43.19 (36.41–48.42)
Region 4	CpG 19	80	58.45	58.99 (49.82–63.95)
	CpG 20	92	53.72	53.12 (44.89–60.07)
	CpG 21	105	64.38	64.83 ± 1.44
	CpG 22	118	59.85	59.85 ± 1.36
Data are shown as mean ± standard error of mean or median (interquartile range, 25–75%).				

**Supplemental Table 5. Difference of DNA methylation level of *FGF6* promoter region between skeletal muscle samples from participants with obesity and normal-weight individuals**				
	CpG sites	Location (distance to TSS)	Obesity–Normal weight (%)	*P*–Value
Region 1	CpG 1	–286	7.68 ± 2.11	0.0009
	CpG 2	–278	7.40 ± 2.79	0.0125
	CpG 3	–270	9.26 ± 2.31	0.0003
	CpG 4	–247	6.58 ± 2.20	0.0054
Region 2	CpG 5	–192	7.93 ± 1.83	0.0001
	CpG 6	–183	7.09 ± 1.61	0.0001
	CpG 7	–179	8.42 ± 1.57	<.0001
	CpG 8	–167	4.09 ± 1.85	0.0343
	CpG 9	–161	4.16 ± 1.83	0.0298
	CpG 10	–144	4.68 ± 1.97	0.0233
	CpG 11	–140	3.43 ± 1.90	0.0807
	CpG 12	–138	2.84 ± 1.57	0.0793
	CpG 13	–107	4.78 ± 2.36	0.0513
	CpG 14	–99	7.14 ± 2.56	0.0093
Region 3	CpG 15	–38	6.49 ± 2.46	0.0126
	CpG 16	–30	6.18 ± 2.74	0.0309
	CpG 17	–7	5.80 ± 1.83	0.0033
	CpG 18	23	5.29 ± 1.96	0.0111
Region 4	CpG 19	80	7.52 ± 2.40	0.0037
	CpG 20	92	8.14 ± 2.42	0.0020
	CpG 21	105	8.69 ± 2.05	0.0002
	CpG 22	118	8.16 ± 1.88	0.0001
Data are shown as mean ± standard error of mean.				

**Supplemental Table 6. Promoter scanning results from JASPAR Database**						
Matrix ID	Name	Score	Relative score	Sequence ID	Strand	Predicted sequence
MA1108.1	MXI1	14.4244	0.967473	seq	+	TGACCACGTGCCT
MA0147.3	MYC	14.3839	0.968142	seq	+	GACCACGTGCCT
MA0058.3	MAX	14.2101	0.996512	seq	+	ACCACGTGCC
MA1653.1	ZNF148	14.0134	0.916812	seq	-	CTCCCCTCCACC
MA0059.1	MAX::MYC	13.9125	0.950276	seq	-	AGGCACGTGGT
MA0052.2	MEF2A	13.7392	0.912815	seq	+	TTCCAAAAATAAGCC
MA0825.1	MNT	13.7024	1	seq	+	ACCACGTGCC
MA0516.1	SP2	13.4473	0.906527	seq	-	GGCCCTCCCCTCCAC
MA0018.3	CREB1	13.223	0.932689	seq	+	GTTGACGTCAGG
MA0018.3	CREB1	13.223	0.932689	seq	-	CCTGACGTCAAC

Supplemental Table 7. Primers for DNA methylation analysis			
Region	CpG sites	Location (distance to TSS)	Sequence
1	1	–286	F1: GGGGAGGTAGTTAGGTTTAAG<br>R1: ACCCCCCTTCTTATTTTTCTCCCT<br>S1: ATGTTATAGTTTTGGATAAGTGA
	2	–278	
	3	–270	
	4	–247	
2	5	–192	F2: GGGTTGTTGAAGTAATTATAAAGATAGG<br>R2: ACTACTAACATAAAACCAAAACCT<br>S2-1: AAAGATAGGTTAAATTAAATATTAT<br>S2-2: AGTAAGAGGGAGAGTTAGAGAG
	6	–183	
	7	–179	
	8	–167	
	9	–161	
	10	–144	
	11	–140	
	12	–138	
	13	–107	
	14	–99	
3	15	–38	F3: AGGTTTTGGTTTTATGTTAGTAGTT<br>R3: AAAACCATCCACCTTACCT<br>S3: ATTTGTTTTTTAAAAATAAGTT
	16	–30	
	17	–7	
	18	23	
4	19	80	F4: GGATGGTTTTGGGATAGAAATTG<br>R4: AAATAACCACTTTCCCAATTCAC<br>S4: GGGATAGAAATTGTTTATTATTATG
	20	92	
	21	105	
	22	118	

F: forward primer, R: reverse primer, S: sequencing primer.

**Supplemental Table 8. Assay ID, and primer sequence for quantitative PCR and plasmids construction**		
Species	Gene	Assay ID or primer sequence
Assay ID		
Human	*FGF6*	Hs00907866_m1
	MYH3	Hs01074230_m1
	XIRP1	Hs00811945_s1
	YTHDC1	Hs00180158_m1
	PPIA	Hs99999904_m1
Primer sequence for quantitative Real-Time PCR		
Mouse	*Mstn*	F: AGTGGATCTAAATGAGGGCAGT
		R: GGAGTACCTCGTGTTTTGTCTC
	Murf1	F: GTGTGAGGTGCCTACTTGCTC
		R: GCTCAGTCTTCTGTCCTTGGA
	Fbxo32	F: TGAACATCATGCAGAGGCTGA
		R: GATCAAACGCTTGCGAATCTG
	Fbxo21	F: TCAATAACCTCAAGGCGTTC
		R: GTTTTGCACACAAGCTCCA
	Igf1	F: CACCTCAGACAGGCATTG
		R: GCTGGGCACGGATAGA
	Fst-total	F: TGCTGCAACACTCTTCCTTG
		R: TGCTGCTACTCTGCCAGTTC
	Fst288	F: CTCTCTCTGCGATGAGCTGTGT
		R: GGCTCAGGTTTTACAGGCAGAT
	Fst315	F: TGCTCTTCTGGCGTGCTTCT
		R: GGAAAGCTGTAGTCCTGGTCTT
	45S Pre-rRNA (ETS)	F: CCAAGTGTTCATGCCACGTG
		R: CGAGCGACTGCCACAAAAA
	45S Pre-rRNA (ITS)	F: CCGGCTTGCCCGATTT
		R: GCCAGCAGGAACGAAACG
	Gapdh	F: CAGCAACTCCCACTCTTCCAC
		R: TGGTCCAGGGTTTCTTACTC
	Tnfα	F: ACGGCATGGATCTCAAAGAC
		R: AGATAGCAAATCGGCTGACG
	Nos2	F: CCAAGCCCTCACCTACTTCC
		R: CTCTGAGGGCTGACACAAGG
	Il1b	F: GCAACTGTTCCTGAACTCAACT
		R: ATCTTTTGGGGTCCGTCAACT
	Il6	F: CCACGGCCTTCCCTACTTC
		R: TTGGGAGTGGTATCCTCTGTGA
	Arg1	F: CTCCAAGCCAAAGTCCTTAGAG
		R: AGGAGCTGTCATTAGGGACATC

	Mrc1	F: GTCAGAACAGACTGCGTGGA
		R: AGGGATCGCCTGTTTTCCAG
	Retnla	F: CTCCACTGTAACGAAGACTC
		R: GCAGTGGTCCAGTCAACGA
	Il10	F: GCTCTTACTGACTGGCATGAG
		R: CGCAGCTCTAGGAGCATGTG
Primer sequence for ChIP-qPCR		
Mouse	ChIP-CRE	F: CTGGGTAAGTGAGCTGGGC
		R: ACGTTCTCTAGCCGGCATG
	ChIP-MYC	F: CAGCCTTCATCTGCCTTCCA
		R: GCCATCCACCTTGCCTCTC
Primers for plasmids construction		
Mouse	*Fgf6* promoter	F: CGGGGTACCAGAAGCCATGGACTGGAGACTTG
		R: GGAAGATCTTCTCAGGCACGTGGTCAGAATT
	Fgf6 promoter del CRE	F: AGTGAGCTGGGCCGGCTTTTTTTTGGGTGGTGATGGGTGA
		R: TCACCCATCACCACCCAAAAAAAAGCCGGCCCAGCTCACT
	Fgf6 promoter del MYC	F: GGCCATTAATTCTGACTTTTTTCCTGAGAAGATCTGCG
		R: CGCAGATCTTCTCAGGAAAAAAGTCAGAATTAATGGCC
F: forward primer, R: reverse primer.		

Supplemental Table 9. Primary antibodies for Western blots

Antibody	Cat No.	Manufacturer	Species
FGF6	#sc-374518	Santa Cruz Biotechnology	Mouse
Puromycin	#MABE343	MilliporeSigma	Mouse
P-4EBP1 $^{Thr37/46}$	#2855	Cell Signaling Technology	Rabbit
4EBP1	#9644	Cell Signaling Technology	Rabbit
P-P70S6K^{Ser434}	#sc-8416	Santa Cruz Biotechnology	Mouse
P70S6K	#2708	Cell Signaling Technology	Rabbit
P-ERK1/2$^{Thr202/Tyr204}$	#9101	Cell Signaling Technology	Rabbit
ERK1/2	#9102	Cell Signaling Technology	Rabbit
P-AKT^{Ser473}	#4060	Cell Signaling Technology	Rabbit
P-AKT^{Thr308}	#13038	Cell Signaling Technology	Rabbit
AKT	#9272	Cell Signaling Technology	Rabbit
P-AMPKα^{Thr172}	#2535	Cell Signaling Technology	Rabbit
AMPK	#2532	Cell Signaling Technology	Rabbit
GAPDH	#2118	Cell Signaling Technology	Rabbit
